# Investigating Mutations to Reduce Huntingtin Aggregation by Increasing Htt-N-Terminal Stability and Weakening Interactions with PolyQ Domain

**DOI:** 10.1155/2016/6247867

**Published:** 2016-12-14

**Authors:** Mohamed R. Smaoui, Cody Mazza-Anthony, Jérôme Waldispühl

**Affiliations:** ^1^Division of Experimental Medicine, Faculty of Medicine, McGill University, Montreal, QC, Canada; ^2^Institut de Recherches Cliniques de Montreal (IRCM), Montreal, QC, Canada; ^3^School of Computer Science, McGill University, Montreal, QC, Canada

## Abstract

Huntington's disease is a fatal autosomal genetic disorder characterized by an expanded glutamine-coding CAG repeat sequence in the huntingtin (Htt) exon 1 gene. The Htt protein associated with the disease misfolds into toxic oligomers and aggregate fibril structures. Competing models for the misfolding and aggregation phenomena have suggested the role of the Htt-N-terminal region and the CAG trinucleotide repeats (polyQ domain) in affecting aggregation propensities and misfolding. In particular, one model suggests a correlation between structural stability and the emergence of toxic oligomers, whereas a second model proposes that molecular interactions with the extended polyQ domain increase aggregation propensity. In this paper, we computationally explore the potential to reduce Htt aggregation by addressing the aggregation causes outlined in both models. We investigate the mutation landscape of the Htt-N-terminal region and explore amino acid residue mutations that affect its structural stability and hydrophobic interactions with the polyQ domain. Out of the millions of 3-point mutation combinations that we explored, the (L4K E12K K15E) was the most promising mutation combination that addressed aggregation causes in both models. The mutant structure exhibited extreme alpha-helical stability, low amyloidogenicity potential, a hydrophobic residue replacement, and removal of a solvent-inaccessible intermolecular side chain that assists oligomerization.

## 1. Background

**Figure 1:**
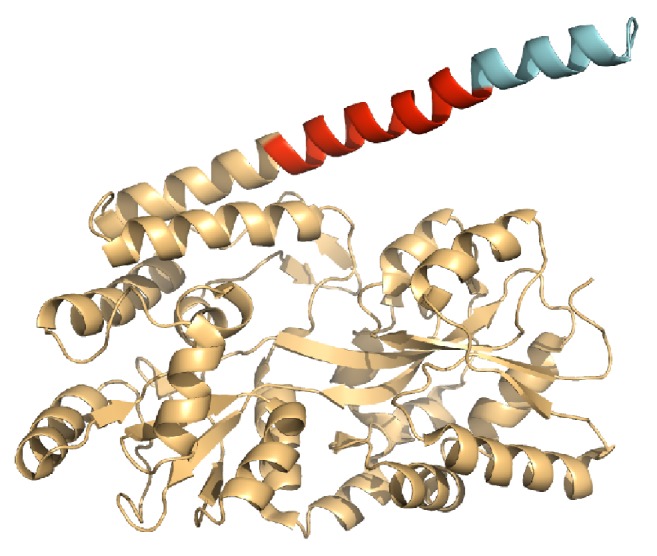
Chain B from PDB 3IO6. Orange residues are positions 1–370 and part of the Htt amino terminal region. The red positions 371–387 are the 17 residues of the Htt-N-terminal region implicated in the formation of amyloids (MATLEKLMKAFESLKSF). The blue residues are GLN repeats attached to the N-terminal region.

Huntington Disease (HD) is a fatal autosomal dominant genetic disorder that is characterized by CAG trinucleotide repeats in the huntingtin (Htt) exon 1 gene [1, 2]. HD is part of the amyloidoses group of diseases characterized by large deposits of amyloid proteins that inflame and weaken cells, leading to their destruction. Parkinson's, Alzheimer's, and diabetes, to name a few, also belong to the same amyloidosis group and have been shown to be greatly aggravated by amyloids [3–5]. These diseases caused or implicated by protein misfolding are usually categorized by the accumulation of insoluble amyloid proteins that form into long fibrils in the body [6]. The build-up of these fibrils causes tissue degradation and appears at the onset of the particular disease. In HD, both the alpha-helical Htt-N-terminal region and the CAG trinucleotide repeats (polyQ domain) are believed to participate in the misfolding process of the huntingtin protein into beta-sheet rich amyloids that aggregate into potentially toxic oligomeric species and fibril structures [7–9]. The wild-type form of Htt is 3144 amino acids in length; however the amyloid form has more CAG repeats causing an extended polyQ domain. The polyQ domain begins at residue 18, as drawn in Figure 1. The rate of aggregation greatly depends on the flanking regions of the polyQ domain. In some cases, the N-terminal domain has been shown to adopt an alpha-helical structure that affects aggregate formation [8] and mutations have been observed to influence fibril formation [10–15].

Htt is found everywhere in human tissues but is significantly expressed in the cerebral cortex of the brain. The homology of Htt is not well known; hence identifying the exact function of the protein has been very difficult. Moreover, the Htt protein interacts with more than 100 different proteins in the body, displaying numerous functions [16]. The ubiquitous Htt in the human body is involved in microtubule machinery and vesicle trafficking [17, 18]. The presence of Htt in the brain of mice has been pivotal to proper development due to its maintenance of ER in neurons [19]. The exact trigger to commence the oligomers and fibril formation is not well defined. Nevertheless, the onset of symptoms is usually attributed to a toxic gain of function by the mutant amyloid [20–22]. This gain of function usually enables a pathway that destabilizes the Htt-N-term to start forming beta-sheets.

There is no known cure for HD and approximately 30,000 people possess the gene that causes HD in the US and Canada. The prevalence of the disease is mainly among Caucasians in a ratio of 5–7 per 100,000 people [23]. As the disease progresses it causes neuronal damage to the brain causing a variety of symptoms including involuntary movements, decreased cognitive function, and psychotic behavior [24]. Current treatments for the disease remain mostly ineffective due to the lack of knowledge surrounding the function of the huntingtin protein [25]. The disease is characterized by intracellular inclusions that form in the GABA-ergic neurons in the brain leading to cell death [26]. The death of these essential neurons leads to uncoordinated movements, erratic body movements, and changes in behavior [27]. Degradation of neuronal pathways leads to dementia and loss of bodily control resulting in injury and eventually full-time medical care. In its last stages, the disease can cause death by triggering failure of most of the body's vital systems.

Stem cell therapy has recently been shown to be a potential treatment for damaged brain cells, allowing for the reversibility of the damages caused by the disease [28]. The disease begins in the central nervous system as large amounts of the healthy form of the Htt protein (native form) misfold and become toxic. The transition between a native and amyloid structure is governed by an amyloidogenic energy barrier that is easier to surpass when the Htt protein is unstable. Hence theoretically, increasing the stability of the Htt protein is one way to potentially decrease the emergence of amyloids and allow for a slower progression of the disease.

Currently, there are two prominent aggregation models outlining how the Htt protein misfolds and aggregates. The first model suggests that the Htt-N-terminal region is the key player behind aggregation, existing normally in a random coil conformation and folding into a helical structure upon interaction with a second Htt-N-terminal to form an oligomer structure [29]. The Htt-N-terminal region is present in helical form in fibrils of huntingtin exon 1, suggesting an involvement in fibril formation, possibly via alpha-helical interactions that create oligomers [8, 30–32]. The Lysine residues (6 and 15) in the Htt-N-terminal region are believed to form an intermolecular side chain interaction and are solvent-inaccessible in the aggregate state [15]. Mutating any of these residues could potentially weaken or break oligomerization. More recently, Arndt et al. strongly confirmed the high degree of helical propensity of the Htt-N-terminal region in solution and observed that some Htt-N-terminal regions turn back on themselves and self-associate with helical bundles [9]. The second model suggests that aggregation of the huntingtin protein occurs as a result of the interaction between the Htt-N-terminal region and huntingtin's extended polyQ domain (the domain cross-talk model) [33, 34]. This model proposes that the hydrophobic residues of the Htt-N-terminal region interact with the polyQ domain and stabilize its structure, driving fibrillization and bypassing the oligomer state.

Computational models have served to increase our understanding of biological processes throughout time. The problem of multiple protein sequence alignment [35], the mapping of molecular evolution [36, 37], and modeling the dynamics of molecules could have only been tackled with the use of computational power [38]. Computational methods assisted analysis on genomic data, mapping of nucleotide and amino acid sequence relationships, exploring protein domains and structures, and storing data sets [39, 40]. The development of computationally intensive techniques and algorithms improved efficient access and use of biological data leading to immediate growth in the fields of drug design, drug discovery, gene finding, and protein structure prediction. The aim of this article is to computationally explore the potential to reduce Htt aggregation by simultaneously addressing the aggregation causes outlined in both models. We investigate the mutation landscape of the Htt-N-terminal region and design potential amino acid residue mutations that can be applied to lower the amyloidogenicity of Htt. We do not promote one aggregation model over the other; rather, we explore mutations that consolidate both aggregation models. To perform this, we outline three procedures. First, we explore the entire stability landscape of the Htt-N-terminal region and identify mutation combinations that significantly increase helical stability to prevent self-association of the Htt-N-terminal region into helical bundles and lower the aggregation propensity suggested by the first model. Second, we analyze the most promising mutation combinations from the previous step in search for mutations that replace hydrophobic residues with charged amino acids to weaken interactions with the polyQ domain outlined in the second model. Finally, we explore any mutation combinations that additionally break the intermolecular side chain between residues 6 and 15, further lowering oligomerization potential. Together, all three procedures simultaneously address the theories behind Htt aggregation proposed by both models. Our results indicate that the mutation combination (L4K E12K K15E) is the most promising mutation that satisfies all three conditions. We confirm with molecular dynamics productions that this mutation combination does not alter native form and results in an Htt-N-terminal mutant structure that exhibits extreme stability and potentially lowers amyloidogenicity. Although not a substitute for experimental studies, computational simulations of proteins can provide insight and test procedures that remain difficult to study experimentally.

## 2. Materials and Methods

In this section, we outline the technical procedure we used to map the mutation landscape of the Htt-N-terminal region and explain in detail how we assess the stability of residues on the protein fragment structure. We performed a brute-force procedure to calculate the stability effect of every possible single-point mutation in the 17 residues of the Htt-N-terminal region and used the results to efficiently estimate the effect of 3-point mutations on the protein fragment.

### 2.1. Exploring Single-Point Mutation Landscape

Algorithm 1 is inspired from Smaoui and Waldispühl [41] and outlines the detailed procedure we used to generate the mutational landscape of the Htt-N-term. Starting with the helical Htt-N-term PDB structure in native form (PDB 3IO6, residues 371–387; see Figure 1), we mutated every residue on the structure backbone into all the other possible 19 canonical amino acids. For each single-point mutation experiment, we compute the Lennard-Jones (LJ), Coulomb, and solvation energy terms. While the LJ and Coulomb measure the electrostatic potential and charges between atoms and solvation measures the interactions with a water surface, together, the sum of the three terms provides a good estimate for the stability of a protein structure, as given by the following:

(1) $$\begin{array}{c} E=Solvation+LJ+Coulomb. \end{array}$$

**Figure 2:**
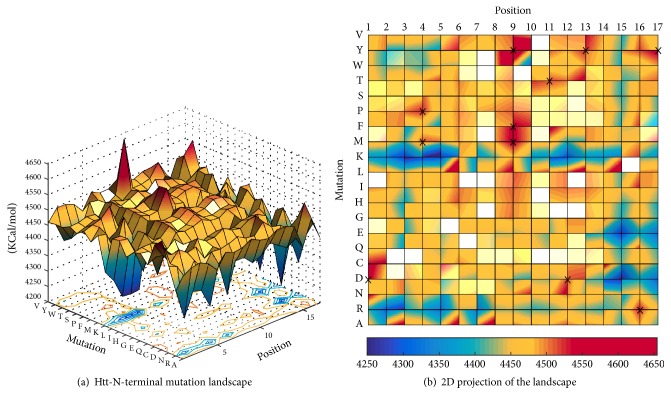
Single-point mutation landscape of the Htt-N-terminal region. Stable mutants have low energies (blue), while destabilizing mutations have the highest energies (red). Energies are in KCal/mol.

Low values indicate stable energetics, while high energy values suggest unfavourable destabilizing interactions. The single-point mutation results generate the 3D landscape plot in Figure 2. The amino acid length of the Htt-N-term makes up 1 dimension, the 20 possible amino acid mutations make up the second dimension, and the energy values produced by a (residue, mutation) pair make up the third dimension.

The LJ and Coulomb terms were calculated using the standard definitions given by the GROMACS 4.5 [42] molecular dynamics package using the GROMOS96 53a6 [43] force field along with the SPC [44] water model. We used a cutoff of 10 Å for van der Waals and short range electrostatic interactions and calculated long range electrostatic interactions using a particle mesh Ewald sum [45, 46].

For every mutation, the sum of LJ, Coulomb, and solvation energies is computed and compared to the nonmutant structure energies as shown in the following:

(2) $$\begin{array}{c} \Delta E=E-E_{0}, \end{array}$$

where *E*_0_ is the energy of the native nonmutant Htt-N-term and *E* is the energy of a native mutant structure.

The Δ*E* value measures the difference in energy caused by introducing a mutation to the Htt-N-term structure. Positive Δ*E* values correlate to a decrease in stability and negative Δ*E* values correlate to an increase in stability.

### 2.2. Solvation Energy

The solvation energy term used to calculate the interactions of our mutant structures with a water surface was computed by AquaSol [47, 48] and solves the following:

(3) Solvation=Fp0,Cdip−F0,0−kBTln⁡1−NACdipa3NACdipa3∫solventdrρdipr,

where *F*_(*p*_0_,*C*_dip_)_ defines the free energy of a protein system defined at dipoles of moment values *p*_0_ and concentration *C*_dip_, *F*_(0,0)_ is the free energy of a protein system with solvent concentration set to zero, *a* ^3^ is the lattice grid size volume of the solvent, *k*_B_ is the Boltzmann constant, *T* is the temperature in Kelvin, and **r** is the surface definition, solvent-accessible surface probe.

The solvation energy is calculated during formation of the protein when it is in water. The tool utilizes the hydrogen bonds between molecules, the pH, and the temperature to efficiently solve the dipolar nonlinear Poisson-Boltzmann-Langevin equation using a fast and detailed dipolar water model. We used AquaSol with the following setup: atomic charges and radii assigned with PDB2PQR using CHARMM force field at neutral pH, a grid of 257 × 257 points spaced by 1 Å, a temperature of 300 K, and a solvent-accessible surface with an Rprobe of 1.4 Å. All hydrogen bonds were optimized. We used a trilinear interpolation protocol for projection of fixed charges on the grid, a lattice grid size for the solvent: *a* = 2.8 Å, and solvent made of dipoles of moment *p*_0_ = 3.00 D at a concentration of *C*_dip_ = 55 M. No salt was added to the solution and small ions (Na^+^, Cl^−^) were used to equilibrate the system when needed. The electrostatic potential was set to zero at the boundaries, and the stopping criteria for residual were sent to 1 · 10^−6^ (when possible).

### 2.3. Performing Mutations

We mutated every residue in the Htt-N-term region into the other 19 canonical amino acids using SCWRL4 [49], a tool that computes the van der Waals forces and hydrogen bond interactions to determine the electron densities of different areas of the protein and predict the side chain conformations to a backbone structure. For every mutation, SCWRL4 fit the new mutant amino acid sequence onto the original Htt-N-terminal backbone to produce a mutant Protein Data Bank (PDB) structure.

### 2.4. Molecular Dynamics Productions

Subsequent to generating the mutant PDB structures for the Htt-N-term, we perform short energy minimization (EM) runs to relax the structures and remove any steric clashes that were introduced by SCWRL4. The difference of the sums of the LJ, Coulomb, and solvation energy terms (2) between the mutants and nonmutant structures was used to rank the stability of the mutations. Mutants that have a Δ*E* that is negative are more stable than the nonmutant structure. Structures that were deemed very stable (low Δ*E*) were then prepared for molecular dynamics (MD) production to formally computationally test structure stability over time.

We used the GROMACS 4.5 [42] molecular simulation package to run molecular dynamics (MD) and energy minimization simulations. Our mutant molecules were solvated in a cubic box (with a minimum distance of 35 Å from any edge of the box to any atom) and neutralized with chloride ions and modeled using the GROMOS96 53a6 force field along with the SPC water model. We used a cutoff of 10 Å for van der Waals and short range electrostatic interactions and calculated long range electrostatic interactions using a particle mesh Ewald sum [45, 46]. Simulations were prepared for a full MD run in both isothermal-isobaric (100 ps) and canonical equilibration (100 ps) ensembles. Temperature and pressure were controlled at 300 K and 1 bar using the velocity rescaling thermostat and the Parrinello-Rahman barostat, respectively. A linear constraint solver was used to keep all bonds at their equilibrium length. Approximately twenty-five million time steps were used with an integration time step of 2 fs to assess any potential turbulence introduced into the molecules by mutations. The system's coordinates were saved every 10 ps for further analysis.

### 2.5. Assessing Structural Deviations

Following EM relaxation, we use MD to compute the stability of the mutant structure over time by analyzing amino acid perturbations using RMSD and RMSF plots. An RMSD plot measures the root mean-square deviations, in angstroms, of the *C*_*α*_ atom positions in protein's residues over a simulation run, whereas the RMSF measures the root mean-square fluctuations, a measure of the deviation between the position of a particle *i* over a simulation run given by

(4) $$\begin{array}{c} RMSF=\frac{1}{T}\overset{T}{\underset{t_{j}=1}{\sum }}{\left(\;x_{i}\left(\;t_{j}\;\right)-{\tilde{x}}_{i}\;\right)}^{2}, \end{array}$$

where *T* is the total simulation time and ${\tilde{x}}_{i}$ is the reference position of particle *i*. Low RMSF at a particular mutation site suggests the absence of local residual instability.

### 2.6. Estimates for Multiple-Point Mutations

The problem of calculating *n*-point mutations (multiple simultaneous single-point mutations) grows exponentially in time and space as *n* increases in size. Even for a reasonable *n* = 3, the number of structures that we need to consider grows to the millions. To circumvent these calculations, we use the data from single-point mutations to estimate the effect of 3-point mutations. This method has been shown to return accurate estimates [41]. We outline the procedure of calculating an approximate Δ*E* for multiple-point mutations in Algorithm 2. The $\Delta \tilde{E}$ estimates are computed by

(5) $$\begin{array}{c} \Delta \tilde{E}=\overset{n}{\underset{i=1}{\sum }}\Delta E\left(\;m_{i},p_{i}\;\right), \end{array}$$

where *m*_*i*_ is the mutation number *i*, *p*_*i*_ is the residue position that the mutation *m*_*i*_ should take effect on, *n* is the total number of desired mutations and the dimension of the landscape, and Δ*E* is the result returned by (2) of mutation *m*_*i*_ on position *p*_*i*_. The estimates are calculated directly by summing values from the Δ*E* table. The estimates might deviate slightly from actual values since they do not take into account pairwise electrostatic and coulomb effects of the mutations with one another.

### 2.7. Predicting Amyloidogenicity

Although the stability results returned by the Htt-N-term landscape predict the effect of mutations on the native helical structure, the landscape does not reveal amyloidogenicity potentials of the mutations. Moreover, the 3-dimensional PDB conformation of the amyloid form of the Htt-N-term is unknown at the present time. Hence, it is difficult to estimate the energy barrier between native and amyloid Htt forms. Nevertheless, we resort to several state-of-the-art computational tools to approximate the 3-point mutations that are both stable and least amyloidogenic, which would result in slower amyloid formation and slower disease progression. In particular, we use Zyggregator [50], TANGO [51], and PASTA [52] to predict the aggregation propensities of the top ranking stable 3-point mutations. The top 3 mutations with best stability and lowest amyloidogenicity were run through an MD production to verify that they conserve the native helical structure of Htt-N-term.

## 3. Results and Discussion

To generate the mutation landscape of the Htt-N-term region of the huntingtin protein we computationally mutated each of the 17 amino acid positions in the N-terminal region to each of the 20 canonical amino acids, as outlined by Algorithm 1. For each mutant structure, we calculated the total energy produced by introducing the respective mutation when computing the electrostatic, solvation, and enthalpy terms in (1). The complete mutation landscape is plotted in Figure 2. Mutations that increase the stability of the structure have the lowest energies, while mutations that destabilize the structure increase its energy. Table S1 (see Table S1 in Supplementary Material available online at http://dx.doi.org/10.1155/2016/6247867) ranks the top 100 mutations in decreasing order of stability. Our results outline that certain single-point mutations lead to an increase in the total energy of the Htt-N-term (mutations with red peaks), while others significantly stabilize the region, potentially lowering amyloidogenicity and aggregation rates of amyloids (mutations with purple nadirs) with respect to the first model of huntingtin aggregation outlined in Section 1. This increase in stability is aimed at preventing self-association of the Htt-N-terminal region into helical bundles [9] and at lowering the potential of aggregation that is not mediated through an oligomeric precursor [53].

### 3.1. Increasing Htt-N-Terminal Stability to Prevent Self-Association and Lower Aggregation Propensity

Three specific mutations in the landscape of Figure 2 introduce a significant increase in structural energy, resulting in a less stable helical form. These three mutations indicate that the Htt-N-term sequence is most likely to destabilize when mutations are applied at the beginning of the sequence (position 1), in the middle (position 9), or at the end (position 17). These corresponding positions show energy spikes in the landscape that can possibly be attributed to the strain that the mutations introduce in the alpha-helical structure. When the Htt-N-term is mutated at position 1 from a Methionine to Aspartic Acid (M1D) it causes an enormous increase in overall energy of the structure. Similarly, mutating the positively charged Lysine at position 9 to a hydrophobic polar Tyrosine (K9Y) and a hydrophobic Phenylalanine to a hydrophobic polar Tyrosine (F17Y) destabilizes the region. In turn, this destabilization could make it easier for the region to break away from its helical form. By decreasing the stability of the Htt-N-term dramatically, the process of aggregation may be increased significantly. Similar to other amyloid proteins in various diseases, the oligomerization of the monomeric forms of the Htt protein is initiated and promoted by the instability in the alpha-helical Htt-N-term region [14, 29, 30, 54]. Apart from the three mutations we mentioned above, the following mutations can also contribute in destabilizing it: L4P, L4M, K9F, K9M, F11T, E12D, S13Y, and S16R. If these mutations find their way into the Htt-N-terminal region, they can potentially promote beta strands to form into long beta-sheets [14, 32, 49].

There are several reasons why certain mutations cause increases in the total energy of the sequence, which include effects caused by structural, electrostatic, and functional groups. Increasing the negative charge of the sequence by phosphorylation of the Htt-N-term region has been shown to decrease fibril formation and disease toxicity [55, 56]. The destabilizing mutation at position 1 increases the total negative charge of the sequence. In conjunction with the electrostatic characteristics of aspartic acid, its steric properties could also contribute to the impairment of the alpha helix assembly which might promote the nucleation of beta-sheets into fibrils. Similarly, the mutations K9Y and F17Y could also have a similar outcome. Tyrosine's high polarity and steric hindrance as a result of its phenol group would make the assembly of the alpha helix extremely difficult. As a result, the rate of fibrillation of Htt proteins could drastically increase leading to higher toxicity in the body.

More importantly, the landscape suggests that various single-point mutations can stabilize the helical N-terminal region. More specifically, Figure 2(b) suggests that four types of mutations can introduce the greatest stability. First, introducing a Lysine (K) into the helical structure can strengthen the bonds of the N-terminal region. This can be seen by the blue dots across the landscape that result from performing a mutation into a K. Similarly, introducing an Arginine (R) into the helical structure also appears to increase stability, regardless of which position it is introduced at (except at position 16). Third, most mutations at positions 15 and 17 result in blue dots, which translates to a relaxation of the structure and increased stability. A complete list of the effect of these mutations on structure energy is ordered in Table S1.

Since our first goal is to explore how to improve the stability of the Htt-N-terminal region to considerably lower amyloidogenicity and aggregation propensities, we can benefit more from mutations by considering the effect of simultaneous multiple-point mutations on the region's stability. We have recently shown that efficiently estimating the structural energy, $\Delta \tilde{E}$, of multiple *n*-point mutations by summing the total effect of the *n*-single-point mutations separately returns accurate estimates so long as the mutations are not all adjacent [41]. We list the top 2-point and 3-point mutations of the Htt-N-terminal region in Tables S2 and S3, respectively, and include in Algorithm 2 the procedure we followed to generate these two lists. Moreover, we capture the best 20 mutation results in Table 1.

Although we can differentiate between the results in Table 1 according to stability, it is unclear which of the top 3-point mutations is the least amyloidogenic. The $\Delta \tilde{E}$ values do not estimate the size of the energy barrier between the native and disordered states of the N-term region. Such an estimate would clearly indicate the effect of the mutations on amyloidogenicity and aggregation propensity. However, this can still be roughly estimated by existing tools. More precisely, we resorted to three tools, Zyggregator [50], TANGO [51], and PASTA [52], to predict the aggregation propensities of the top 3-point mutations. The results of each of the tools are listed in Tables 2 [Table tab3]–4. The results returned by Zyggregator in Table 2 do not suggest a difference in amyloidogenicity in the 20 sequences. The beta-sheet propensities are similar, the alpha-helical propensities only differ slightly, and the intrinsic aggregation propensities are within close range. The results returned by TANGO in Table 3 suggest that result M16 (L4K E12K K15E) has the lowest amyloidogenic potential, a relatively high helical potential, and one of the lowest beta-sheet propensities. The results returned by PASTA in Table 4 favor result M7 (T3K E5K K15E). Result M7 has the highest percentage of residues predicted to be in a helix state and is the most stable out of the set, resulting in the lowest aggregation potential. The stability results in Table 1 satisfy conditions from the first model of aggregation, suggesting that M16 and M7 are potentially the most stable Htt-N-terminal mutant forms.

### 3.2. Weakening Interactions between the Htt-N-Terminal Region and the PolyQ Domain

The second step of this multiobjective approach is to analyze which of the mutation combinations has likely weaken interactions with the polyQ domain, satisfying the condition set by the second model of aggregation (see Section 1). For this procedure, we considered mutations that replace hydrophobic residues on the Htt-N-term with charged ones. In the domain cross-talk model, the hydrophobic residues are responsible for playing a part in stabilizing the polyQ domain. Altering hydrophobic residues to charged residues adds strain to the Htt-N-term and affects the stability and length of the polyQ domain, altering aggregation. From Table 1, we find that most mutation combinations in our list already substitute a hydrophobic amino acid with a charged reside (M1, M2, M4, M5, M6, M8, M11, M14, M15, M16, M18, and M20). Although the Htt-N-terminal region contains several hydrophobic residues, it has been shown that removing just one of the hydrophobic amino acids can effectively weaken the interactions with the polyQ domain and potentially reduce overall aggregation [9]. We acknowledge that we cannot quantitatively model the interactions of the mutations with the polyQ domain without a good PDB representation; however, we are relying on the theoretical understanding behind the interactions of the hydrophobic residues and the polyQ domain to conclude that the charged residues with weaken interactions.

### 3.3. Breaking the Solvent-Inaccessible Intermolecular Side Chain of the Htt-N-Terminal Region

In addition to satisfying the first two conditions of helical stability and hydrophilic substitution, the M16 combination luckily also contains a K15E substitution. Substituting the Lysine residue at position 15 breaks the solvent-inaccessible intermolecular side chain interaction that contributes to oligomerization [9].

**Figure 3:**
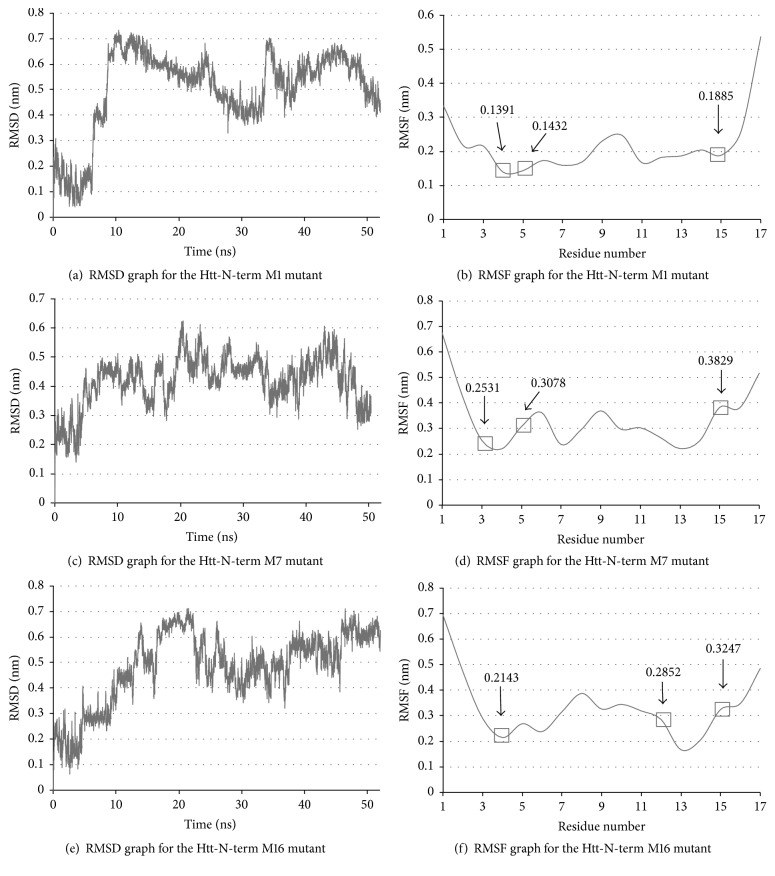
MD results of mutants 1, 7, and 16. (a) A 52 ns RMSD plot of mutant (L4K E5K K15D) and (b) the RMSF plot of the same mutant. (c) A 50 ns RMSD plot of mutant (T3K E5K K15E) and (d) the RMSF values of the same mutant. (e) a 52 ns RMSD plot of mutant (L4K E12K K15E) and (f) the RMSF values of the same mutant. All structures appear to be stable and conserved.

Since we are introducing three mutations to the Htt-N-terminal structure and lowering the stability by a large factor, it remains to check that the mutations do not alter the 3D helical conformation of the region. To check this, we performed a complete molecular dynamics simulation of 50 nanoseconds on the most stable mutants from Table 1 (M1, M7, and M16) and found that indeed the Htt-N-terminal region maintains its stability in all three structures. We report the results of the RMSD and RMSF graphs of each mutant structure in Figure 3. Figures 3(a), 3(c), and 3(e) plot the RMSD fluctuations over time and Figures 3(b), 3(d), and 3(f) plot the RMSF graphs indicating the stability of the mutated residue positions during the respective MD run. The RMSF graphs show that the regions around the mutated residues are relatively stable and the RMSD graphs all report values less than 1 nm. While all the three mutant structures appear to exhibit good stability, we observe that mutant M16 shows less RMSD variations than M1 and more stable RMSF values than M7 at the mutated residues (positions 4, 5, and 15). For all the reasons we explored above, we believe that the M16 mutant (L4K E12K K15E) is the best mutation candidate to lower the amyloidogenicity of Htt in both models of huntingtin aggregation.

## 4. Conclusion

The Htt protein is a huge protein that contains 3144 amino acids. One limitation of the study is that we did not consider the entire structure in our energy predictions. The simulation run-time would have been excessive and it was therefore not possible to conduct MD simulations and stability analysis on the entire protein. However, many studies have consistently reported valid findings by only examining the N-term region of Htt and considered it the most important component to study in the progression of Huntington's disease [11–14, 47, 57].

The two models of huntingtin aggregation describe the role of the Htt-N-terminal region in (1) utilizing alpha-helical interactions to create oligomers and (2) the role of its hydrophobic residues in stabilizing the polyQ domain and exciting aggregation. We explored the mutation landscape of this structure by mutating amino acid residues and calculating the resulting changes in total energy. Our goal was to probe the entire mutation landscape of the Htt-N-terminal region to identify mutations that would lower the aggregation propensities outlined in both Htt models. By identifying critical maxima and minima points in the landscape, experimentalists have a smaller subset of possibilities to test for mutants that change HD progression rates.

Exploring the entire stability landscape of the Htt-N-terminal region enabled us to identify mutation combinations that significantly increase helical stability to prevent self-association of the Htt-N-terminal region into helical bundles and lower the aggregation propensity suggested by the first model (see Section 1 for model descriptions). We investigated the mutation landscape of the Htt-N-term to unravel unstable regions characterized by high energy values while identifying regions that can further stabilize the Htt-N-term. By narrowing our search space for lowest amyloidogenic potential mutations, we calculated the $\Delta \tilde{E}$ for multiple-point mutations (2-point and 3-point). We focused on mutants that possessed the lowest energies and used state-of-the-art tools to predict which of those mutations have promising low amyloidogenic propensities. We analyzed the most promising mutation combinations that additionally replace hydrophobic residues with charged amino acids to weaken interactions with the polyQ domain to lower the aggregation resulting from interaction with the polyQ domain. Out of the millions of 3-point mutations that we considered, the (L4K E12K K15E) mutant exhibited extreme stability, low amyloidogenicity, hydrophobic replacement, and removal of the solvent-inaccessible intermolecular side chain that assists oligomerization. The results we explored computationally can serve as possibilities that experimentalists can potentially utilize.

## Supplementary Material

Exploring the mutational landscape of the htt-n-term region of the Huntingtin protein for critical 1-point, 2-point, and 3-point mutations.

## Figures and Tables

**Algorithm 1:**
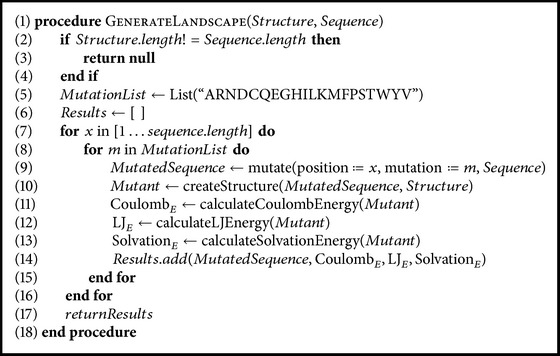
Generating the mutational landscapes of the Htt-N-term.

**Algorithm 2:**
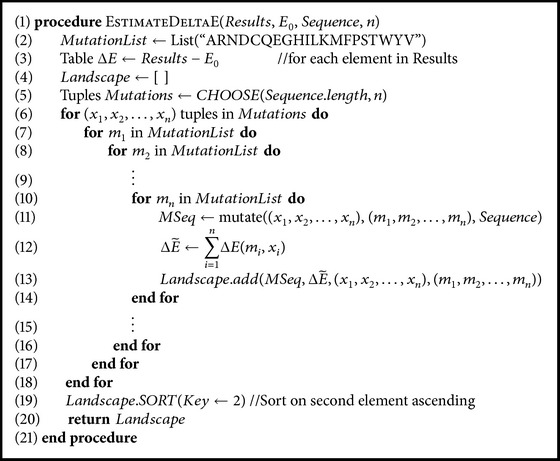
Generating $\Delta \tilde{E}$ for *n*-point mutations.

**Table 1:** The top 3-point mutations in Htt-N-term with lowest $\Delta \tilde{E}$ values.

Mutant	Sequence	$\Delta \tilde{E}$	Mutations
M1	- - - KK - - - - - - - - - D - -	−668.3	L4K E5K K15D
M2	- - KK - - - - - - - - - - D - -	−667.7	T3K L4K K15D
M3	- - K-K - - - - - - - - - D - -	−664.9	T3K E5K K15D
M4	- - - KK - - - - - - - - - E - -	−649	L4K E5K K15E
M5	- - KK - - - - - - - - - - E - -	−648.4	T3K L4K K15E
M6	- - - K - - - - - - - K - - D - -	−646.7	L4K E12K K15D
M7	- - K-K - - - - - - - - - E - -	−645.7	T3K E5K K15E
M8	- - KKK - - - - - - - - - - - -	−645.6	T3K L4K E5K
M9	- - - - K - - - - - - K - - D - -	−644	E5K E12K K15D
M10	- - K - - - - - - - - K - - D - -	−643.4	T3K E12K K15D
M11	- - - K - - - - - - - - - - D-D	−637.3	L4K K15D F17D
M12	- - - - K - - - - - - - - - D-D	−634.5	E5K K15D F17D
M13	- - K - - - - - - - - - - - D-D	−633.9	T3K K15D F17D
M14	- - - K - - - - - - - - - - D-E	−629.4	L4K K15D F17E
M15	-R-K - - - - - - - - - - D - -	−629.2	A2R L4K K15D
M16	- - - K - - - - - - - K - - E - -	−627.5	L4K E12K K15E
M17	- - - - K - - - - - - - - - D-E	−626.6	E5K K15D F17E
M18	-R - - K - - - - - - - - - D - -	−626.5	A2R E5K K15D
M19	- - K - - - - - - - - - - - D-E	−626	T3K K15D F17E
M20	- RK - - - - - - - - - - - D - -	−625.8	A2R T3K K15D

**Table 2:** Aggregation potentials of top 20 results for Htt-N-term 3-point mutations computed by Zyggregator.

Mutant	Hydrophobicity	Charge	Beta-sheet propensity	Alpha-helical propensity	Aggregation propensity
Native	4.91	1	74.04	75.00	−5.01
M1	10.40	2	73.87	74.39	−5.37
M2	12.31	1	73.71	75.01	−5.37
M3	7.58	2	73.87	75.04	−5.49
M4	9.50	2	73.87	74.87	−5.50
M5	11.41	1	73.71	75.49	−5.50
M6	10.40	2	73.87	74.39	−5.37
M7	6.68	2	73.87	75.52	−5.62
M8	11.13	5	73.71	75.30	−6.70
M9	5.67	3	74.04	74.42	−5.48
M10	7.58	2	73.87	75.04	−5.49
M11	16.62	−1	73.58	74.24	−5.30
M12	11.89	0	73.74	74.27	−4.78
M13	13.80	−1	73.58	74.89	−5.42
M14	15.72	−1	73.58	74.72	−5.43
M15	14.88	1	74.10	74.67	−5.24
M16	9.50	2	73.87	74.87	−5.50
M17	10.99	0	73.74	74.75	−4.91
M18	10.15	2	74.26	74.70	−5.35
M19	12.90	−1	73.58	75.37	−5.55
M20	12.06	1	74.10	75.33	−5.36

**Table 3 tab3:** Aggregation potentials of top 20 results for Htt-N-term 3-point mutations computed by TANGO.

Mutant	Amylo	Turn	Helix	Beta
Native	0.55	3.17	28.74	15.36
M1	4.18	6.31	1.26	20.41
M2	0.57	6.38	0.00	18.00
M3	0.57	6.16	0.00	20.56
M4	4.21	3.67	1.26	15.58
M5	0.57	3.75	0.00	13.17
M6	0.00	6.11	16.82	19.46
M7	0.57	3.53	0.00	15.73
M8	0.57	3.64	0.00	11.63
M9	0.00	5.87	9.52	26.75
M10	0.00	5.95	8.95	21.72
M11	0.56	5.92	7.97	19.19
M12	4.16	5.65	6.32	26.40
M13	0.56	5.75	1.85	21.45
M14	0.56	5.80	7.97	19.42
M15	0.08	6.54	0.00	20.13
M16	0.00	3.49	17.05	14.66
M17	4.16	5.53	6.32	26.63
M18	0.95	6.24	0.00	27.17
M19	0.57	5.63	1.85	21.68
M20	0.34	6.25	1.06	23.01

The Amylo column of the results returned by TANGO suggests that mutant M16 has the least amyloidogenicity.

**Table 4:** Aggregation potentials of top 20 results for Htt-N-term 3-point mutations computed by PASTA.

Mutant	Best energy	% *α*-helix	% coil
Native	−1.12	76.47	23.53
M1	−0.48	70.59	29.41
M2	−0.48	76.47	23.53
M3	−0.90	76.47	23.53
M4	−0.82	70.59	29.41
M5	−0.82	76.47	23.53
M6	−1.07	70.59	29.41
M7	−1.50	82.35	17.65
M8	−1.00	76.47	23.53
M9	−0.68	76.47	23.53
M10	−1.12	76.47	23.53
M11	−1.07	64.71	35.29
M12	−0.90	76.47	23.53
M13	−1.12	76.47	23.53
M14	−1.07	64.71	35.29
M15	−0.91	70.59	29.41
M16	−1.07	70.59	29.41
M17	−0.90	76.47	23.53
M18	−0.90	76.47	23.53
M19	−1.12	76.47	23.53
M20	−1.12	76.47	23.53

Mutant M7 results in the lowest energy, highest %  *α*-helix, and lowest % coil.
