# Supplementary material for: Investigating Mutations to Reduce Huntingtin Aggregation by Increasing Htt-N-Terminal Stability and Weakening Interactions with PolyQ Domain
Source: Comput Math Methods Med. 2016 Dec 14;2016:6247867. doi: 10.1155/2016/6247867 (PMC5206856; doi:10.1155/2016/6247867)
Supplement: Supplementary file 1 — Exploring the mutational landscape of the htt-n-term region of the Huntingtin protein for critical 1-point, 2-point, and 3-point mutations. [file 6247867.f1.pdf]

Supporting Information

Table S1: The top 100 1-point mutations in htt-n-term with lowest ΔE values

No.	Sequence	ΔE	Mutations
1	--------------D--	-236.6	K15D
2	--------------E--	-217.3	K15E
3	---K-------------	-217.2	L4K
4	----K------------	-214.5	E5K
5	--K--------------	-213.9	T3K
6	-----------K-----	-192.9	E12K
7	----------------D	-183.5	F17D
8	----------------E	-175.6	F17E
9	-R---------------	-175.4	A2R
10	----R------------	-170.5	E5R
11	------R----------	-155.3	L7R
12	-K---------------	-150.2	A2K
13	------K----------	-123.6	L7K
14	-------------K---	-123.4	L14K
15	------------K----	-113.7	S13K
16	---------K-------	-112.4	A10K
17	K----------------	-111.7	M1K
18	---R-------------	-107.9	L4R
19	--H--------------	-102.4	T3H
20	--R--------------	-99.1	T3R
21	---------------E-	-94.4	S16E
22	--E--------------	-93.7	T3E
23	----------------H	-93.3	F17H
24	---------------D-	-93.2	S16D
25	----------K------	-89.1	F11K
26	-------------E---	-88.7	L14E
27	--------------Q--	-87.6	K15Q
28	-Y---------------	-87.2	A2Y
29	----------------N	-85.2	F17N
30	---------R-------	-85.2	A10R
31	--------------G--	-84.7	K15G
32	---W-------------	-84.2	L4W
33	--------------Y--	-81.2	K15Y
34	-------------D---	-80.9	L14D
35	--------------A--	-80.9	K15A
36	-------------R---	-80.7	L14R
37	--------------T--	-80.1	K15T
38	----------------Q	-78.4	F17Q
39	--------------H--	-78	K15H
40	-----------M-----	-77.6	E12M
41	--------------S--	-77.1	K15S

Continued on next page

Table S1 – continued from previous page

No.	Sequence	ΔE	Mutations
42	--M--------------	-76.4	T3M
43	R----------------	-75.2	M1R
44	--------------L--	-73.6	K15L
45	----------------S	-72.6	F17S
46	--------------P--	-72.3	K15P
47	---------------K-	-72.3	S16K
48	----------------A	-71.3	F17A
49	----------R------	-71.2	F11R
50	---Y-------------	-71.2	L4Y
51	-W---------------	-70.2	A2W
52	----------E------	-69.9	F11E
53	----------------C	-69.8	F17C
54	----------------G	-69.4	F17G
55	-------K---------	-69.2	M8K
56	--------D--------	-68.9	K9D
57	----------------P	-67.8	F17P
58	--N--------------	-66.6	T3N
59	----------------K	-66.4	F17K
60	--------------C--	-65.9	K15C
61	--Y--------------	-63.8	T3Y
62	--------------V--	-62.1	K15V
63	------------R----	-61.6	S13R
64	--------------R--	-61.4	K15R
65	----N------------	-59.7	E5N
66	--W--------------	-58.5	T3W
67	----------D------	-57.9	F11D
68	--------R--------	-56.6	K9R
69	-------R---------	-56.2	M8R
70	--------E--------	-51.2	K9E
71	-------E---------	-51	M8E
72	----Q------------	-50.8	E5Q
73	--I--------------	-49.8	T3I
74	----------------M	-47.6	F17M
75	--------------W--	-47.2	K15W
76	------------D----	-47	S13D
77	--F--------------	-46.8	T3F
78	----C------------	-46.2	E5C
79	----S------------	-45.9	E5S
80	----W------------	-45.8	E5W
81	--------------F--	-45.7	K15F
82	----G------------	-45	E5G
83	--L--------------	-44.2	T3L
84	-----D-----------	-43.9	K6D
85	----------------I	-43	F17I
86	-----R-----------	-42.9	K6R
87	---------------F-	-41.9	S16F
88	-----------R-----	-41.9	E12R
89	----A------------	-41.9	E5A
90	----M------------	-41.7	E5M
91	----------------V	-41.1	F17V
92	-----------W-----	-40.1	E12W
93	----F------------	-39.4	E5F

Continued on next page

Table S1 – continued from previous page

No.	Sequence	ΔE	Mutations
94	--------------N--	-39.4	K15N
95	-------D---------	-38.9	M8D
96	---------E-------	-36.9	A10E
97	----T------------	-36.4	E5T
98	----------------R	-36.1	F17R
99	--------------I--	-34.3	K15I
100	----P------------	-33.8	E5P

Table S2: The top 100 2-point mutations in htt-n-term with lowest ΔE values

No.	Sequence	$\Delta\tilde{E}$	Mutations
1	---K----------D--	-453.8	L4K K15D
2	----K---------D--	-451.1	E5K K15D
3	--K-----------D--	-450.4	T3K K15D
4	---K----------E--	-434.5	L4K K15E
5	----K---------E--	-431.8	E5K K15E
6	---KK------------	-431.7	L4K E5K
7	--K-----------E--	-431.2	T3K K15E
8	--KK-------------	-431.1	T3K L4K
9	-----------K--D--	-429.5	E12K K15D
10	--K-K------------	-428.4	T3K E5K
11	--------------D-D	-420	K15D F17D
12	--------------D-E	-412.1	K15D F17E
13	-R------------D--	-412	A2R K15D
14	-----------K--E--	-410.2	E12K K15E
15	---K-------K-----	-410.2	L4K E12K
16	----K------K-----	-407.4	E5K E12K
17	----R---------D--	-407	E5R K15D
18	--K--------K-----	-406.8	T3K E12K
19	--------------E-D	-400.8	K15E F17D
20	---K------------D	-400.7	L4K F17D
21	----K-----------D	-398	E5K F17D
22	--K-------------D	-397.4	T3K F17D
23	--------------E-E	-392.9	K15E F17E
24	---K------------E	-392.8	L4K F17E
25	-R------------E--	-392.7	A2R K15E
26	-R-K-------------	-392.6	A2R L4K
27	------R-------D--	-391.8	L7R K15D
28	----K-----------E	-390.1	E5K F17E
29	-R--K------------	-389.9	A2R E5K
30	--K-------------E	-389.5	T3K F17E
31	-RK--------------	-389.3	A2R T3K
32	----R---------E--	-387.8	E5R K15E
33	---KR------------	-387.7	L4K E5R
34	-K------------D--	-386.8	A2K K15D
35	--K-R------------	-384.4	T3K E5R
36	-----------K----D	-376.4	E12K F17D
37	------R-------E--	-372.6	L7R K15E
38	---K--R----------	-372.5	L4K L7R
39	----K-R----------	-369.8	E5K L7R
40	--K---R----------	-369.2	T3K L7R
41	-----------K----E	-368.5	E12K F17E
42	-R---------K-----	-368.3	A2R E12K
43	-K------------E--	-367.5	A2K K15E
44	-K-K-------------	-367.4	A2K L4K
45	-K--K------------	-364.7	A2K E5K
46	-KK--------------	-364.1	A2K T3K
47	----R------K-----	-363.4	E5R E12K
48	------K-------D--	-360.2	L7K K15D
49	-------------KD--	-359.9	L14K K15D
50	-R--------------D	-358.9	A2R F17D

Continued on next page

Table S2 – continued from previous page

No.	Sequence	$\Delta\tilde{E}$	Mutations
51	----R-----------D	-354	E5R F17D
52	-R--------------E	-351	A2R F17E
53	------------K-D--	-350.2	S13K K15D
54	---------K----D--	-349	A10K K15D
55	K-------------D--	-348.2	M1K K15D
56	------R----K-----	-348.2	L7R E12K
57	----R-----------E	-346.1	E5R F17E
58	-R--R------------	-345.9	A2R E5R
59	---R----------D--	-344.5	L4R K15D
60	-K---------K-----	-343.2	A2K E12K
61	------K-------E--	-340.9	L7K K15E
62	---K--K----------	-340.9	L4K L7K
63	-------------KE--	-340.7	L14K K15E
64	---K---------K---	-340.6	L4K L14K
65	--H-----------D--	-339	T3H K15D
66	------R---------D	-338.7	L7R F17D
67	----K-K----------	-338.1	E5K L7K
68	----K--------K---	-337.9	E5K L14K
69	--K---K----------	-337.5	T3K L7K
70	--K----------K---	-337.2	T3K L14K
71	--R-----------D--	-335.6	T3R K15D
72	-K--------------D	-333.7	A2K F17D
73	------------K-E--	-331	S13K K15E
74	--------------DE-	-330.9	K15D S16E
75	---K--------K----	-330.9	L4K S13K
76	------R---------E	-330.9	L7R F17E
77	-R----R----------	-330.7	A2R L7R
78	--E-----------D--	-330.3	T3E K15D
79	--------------D-H	-329.9	K15D F17H
80	--------------DD-	-329.8	K15D S16D
81	---------K----E--	-329.7	A10K K15E
82	---K-----K-------	-329.6	L4K A10K
83	K-------------E--	-329	M1K K15E
84	K--K-------------	-328.9	M1K L4K
85	----K-------K----	-328.2	E5K S13K
86	--K---------K----	-327.5	T3K S13K
87	----K----K-------	-326.9	E5K A10K
88	--K------K-------	-326.3	T3K A10K
89	K---K------------	-326.2	M1K E5K
90	-K--------------E	-325.8	A2K F17E
91	----R-R----------	-325.8	E5R L7R
92	----------K---D--	-325.7	F11K K15D
93	K-K--------------	-325.6	M1K T3K
94	---R----------E--	-325.2	L4R K15E
95	-------------ED--	-325.2	L14E K15D
96	-Y------------D--	-323.8	A2Y K15D
97	---RK------------	-322.4	L4R E5K
98	--KR-------------	-321.8	T3K L4R
99	--------------D-N	-321.8	K15D F17N
100	---------R----D--	-321.7	A10R K15D

Table S3: The top 1000 3-point mutations in htt-n-term with lowest $\Delta\tilde{E}$ values

No.	Sequence	$\Delta\tilde{E}$	Mutations
1	---KK---------D--	-668.3	L4K E5K K15D
2	--KK----------D--	-667.7	T3K L4K K15D
3	--K-K---------D--	-664.9	T3K E5K K15D
4	---KK---------E--	-649	L4K E5K K15E
5	--KK----------E--	-648.4	T3K L4K K15E
6	---K-------K--D--	-646.7	L4K E12K K15D
7	--K-K---------E--	-645.7	T3K E5K K15E
8	--KKK------------	-645.6	T3K L4K E5K
9	----K------K--D--	-644	E5K E12K K15D
10	--K--------K--D--	-643.4	T3K E12K K15D
11	---K----------D-D	-637.3	L4K K15D F17D
12	----K---------D-D	-634.5	E5K K15D F17D
13	--K-----------D-D	-633.9	T3K K15D F17D
14	---K----------D-E	-629.4	L4K K15D F17E
15	-R-K----------D--	-629.2	A2R L4K K15D
16	---K-------K--E--	-627.5	L4K E12K K15E
17	----K---------D-E	-626.6	E5K K15D F17E
18	-R--K---------D--	-626.5	A2R E5K K15D
19	--K-----------D-E	-626	T3K K15D F17E
20	-RK-----------D--	-625.8	A2R T3K K15D
21	----K------K--E--	-624.7	E5K E12K K15E
22	---KK------K-----	-624.7	L4K E5K E12K
23	---KR---------D--	-624.3	L4K E5R K15D
24	--K--------K--E--	-624.1	T3K E12K K15E
25	--KK-------K-----	-624	T3K L4K E12K
26	--K-K------K-----	-621.3	T3K E5K E12K
27	--K-R---------D--	-620.9	T3K E5R K15D
28	---K----------E-D	-618	L4K K15E F17D
29	----K---------E-D	-615.3	E5K K15E F17D
30	---KK-----------D	-615.2	L4K E5K F17D
31	--K-----------E-D	-614.7	T3K K15E F17D
32	--KK------------D	-614.6	T3K L4K F17D
33	-----------K--D-D	-613	E12K K15D F17D
34	--K-K-----------D	-611.9	T3K E5K F17D
35	---K----------E-E	-610.1	L4K K15E F17E
36	-R-K----------E--	-609.9	A2R L4K K15E
37	---K--R-------D--	-609.1	L4K L7R K15D
38	----K---------E-E	-607.4	E5K K15E F17E
39	---KK-----------E	-607.3	L4K E5K F17E
40	-R--K---------E--	-607.2	A2R E5K K15E
41	-R-KK------------	-607.1	A2R L4K E5K
42	--K-----------E-E	-606.8	T3K K15E F17E
43	--KK------------E	-606.7	T3K L4K F17E
44	-RK-----------E--	-606.6	A2R T3K K15E
45	-RKK-------------	-606.5	A2R T3K L4K
46	----K-R-------D--	-606.3	E5K L7R K15D
47	--K---R-------D--	-605.7	T3K L7R K15D
48	-----------K--D-E	-605.1	E12K K15D F17E
49	---KR---------E--	-605	L4K E5R K15E
50	-R---------K--D--	-604.9	A2R E12K K15D

Continued on next page

Table S3 – continued from previous page

No.	Sequence	$\Delta\tilde{E}$	Mutations
51	-K-K----------D--	-604	A2K L4K K15D
52	--K-K-----------E	-604	T3K E5K F17E
53	-RK-K------------	-603.8	A2R T3K E5K
54	--K-R---------E--	-601.7	T3K E5R K15E
55	--KKR------------	-601.6	T3K L4K E5R
56	-K--K---------D--	-601.3	A2K E5K K15D
57	-KK-----------D--	-600.7	A2K T3K K15D
58	----R------K--D--	-600	E5R E12K K15D
59	-R------------D-D	-595.4	A2R K15D F17D
60	-----------K--E-D	-593.7	E12K K15E F17D
61	---K-------K----D	-593.6	L4K E12K F17D
62	----K------K----D	-590.9	E5K E12K F17D
63	----R---------D-D	-590.5	E5R K15D F17D
64	--K--------K----D	-590.3	T3K E12K F17D
65	---K--R-------E--	-589.8	L4K L7R K15E
66	-R------------D-E	-587.5	A2R K15D F17E
67	----K-R-------E--	-587.1	E5K L7R K15E
68	---KK-R----------	-587	L4K E5K L7R
69	--K---R-------E--	-586.5	T3K L7R K15E
70	--KK--R----------	-586.4	T3K L4K L7R
71	-----------K--E-E	-585.8	E12K K15E F17E
72	---K-------K----E	-585.8	L4K E12K F17E
73	-R---------K--E--	-585.6	A2R E12K K15E
74	-R-K-------K-----	-585.6	A2R L4K E12K
75	------R----K--D--	-584.8	L7R E12K K15D
76	-K-K----------E--	-584.7	A2K L4K K15E
77	--K-K-R----------	-583.7	T3K E5K L7R
78	----K------K----E	-583	E5K E12K F17E
79	-R--K------K-----	-582.8	A2R E5K E12K
80	----R---------D-E	-582.6	E5R K15D F17E
81	-R--R---------D--	-582.5	A2R E5R K15D
82	--K--------K----E	-582.4	T3K E12K F17E
83	-RK--------K-----	-582.2	A2R T3K E12K
84	-K--K---------E--	-582	A2K E5K K15E
85	-K-KK------------	-582	A2K L4K E5K
86	-KK-----------E--	-581.4	A2K T3K K15E
87	-KKK-------------	-581.3	A2K T3K L4K
88	----R------K--E--	-580.7	E5R E12K K15E
89	---KR------K-----	-580.7	L4K E5R E12K
90	-K---------K--D--	-579.7	A2K E12K K15D
91	-KK-K------------	-578.6	A2K T3K E5K
92	---K--K-------D--	-577.4	L4K L7K K15D
93	--K-R------K-----	-577.3	T3K E5R E12K
94	---K---------KD--	-577.1	L4K L14K K15D
95	-R------------E-D	-576.2	A2R K15E F17D
96	-R-K------------D	-576.1	A2R L4K F17D
97	------R-------D-D	-575.3	L7R K15D F17D
98	----K-K-------D--	-574.7	E5K L7K K15D
99	----K--------KD--	-574.4	E5K L14K K15D
100	--K---K-------D--	-574.1	T3K L7K K15D
101	--K----------KD--	-573.8	T3K L14K K15D
102	-R--K-----------D	-573.4	A2R E5K F17D

Continued on next page

Table S3 – continued from previous page

No.	Sequence	$\Delta\tilde{E}$	Mutations
103	-RK-------------D	-572.8	A2R T3K F17D
104	----R---------E-D	-571.3	E5R K15E F17D
105	---KR-----------D	-571.2	L4K E5R F17D
106	-K------------D-D	-570.2	A2K K15D F17D
107	-R------------E-E	-568.3	A2R K15E F17E
108	-R-K------------E	-568.2	A2R L4K F17E
109	--K-R-----------D	-567.8	T3K E5R F17D
110	---K--------K-D--	-567.4	L4K S13K K15D
111	------R-------D-E	-567.4	L7R K15D F17E
112	-R----R-------D--	-567.2	A2R L7R K15D
113	---K-----K----D--	-566.2	L4K A10K K15D
114	------R----K--E--	-565.5	L7R E12K K15E
115	-R--K-----------E	-565.5	A2R E5K F17E
116	K--K----------D--	-565.5	M1K L4K K15D
117	---K--R----K-----	-565.4	L4K L7R E12K
118	-RK-------------E	-564.9	A2R T3K F17E
119	----K-------K-D--	-564.7	E5K S13K K15D
120	--K---------K-D--	-564.1	T3K S13K K15D
121	----K----K----D--	-563.5	E5K A10K K15D
122	----R---------E-E	-563.4	E5R K15E F17E
123	---KR-----------E	-563.3	L4K E5R F17E
124	-R--R---------E--	-563.2	A2R E5R K15E
125	-R-KR------------	-563.1	A2R L4K E5R
126	--K------K----D--	-562.9	T3K A10K K15D
127	K---K---------D--	-562.7	M1K E5K K15D
128	----K-R----K-----	-562.7	E5K L7R E12K
129	-K------------D-E	-562.4	A2K K15D F17E
130	----R-R-------D--	-562.3	E5R L7R K15D
131	K-K-----------D--	-562.1	M1K T3K K15D
132	--K---R----K-----	-562.1	T3K L7R E12K
133	-K---------K--E--	-560.5	A2K E12K K15E
134	-K-K-------K-----	-560.4	A2K L4K E12K
135	--K-R-----------E	-560	T3K E5R F17E
136	-RK-R------------	-559.8	A2R T3K E5R
137	---RK---------D--	-559	L4R E5K K15D
138	--KR----------D--	-558.4	T3K L4R K15D
139	---K--K-------E--	-558.2	L4K L7K K15E
140	---K---------KE--	-557.9	L4K L14K K15E
141	-K--K------K-----	-557.7	A2K E5K E12K
142	-K--R---------D--	-557.3	A2K E5R K15D
143	-KK--------K-----	-557	A2K T3K E12K
144	--HK----------D--	-556.2	T3H L4K K15D
145	------R-------E-D	-556	L7R K15E F17D
146	---K--R---------D	-556	L4K L7R F17D
147	----K-K-------E--	-555.4	E5K L7K K15E
148	---KK-K----------	-555.4	L4K E5K L7K
149	----K--------KE--	-555.2	E5K L14K K15E
150	---KK--------K---	-555.1	L4K E5K L14K
151	--K---K-------E--	-554.8	T3K L7K K15E
152	--KK--K----------	-554.7	T3K L4K L7K
153	--K----------KE--	-554.5	T3K L14K K15E
154	--KK---------K---	-554.5	T3K L4K L14K

Continued on next page

Table S3 – continued from previous page

No.	Sequence	$\Delta\tilde{E}$	Mutations
155	--H-K---------D--	-553.5	T3H E5K K15D
156	----K-R---------D	-553.2	E5K L7R F17D
157	------K----K--D--	-553.1	L7K E12K K15D
158	-----------K-KD--	-552.9	E12K L14K K15D
159	--RK----------D--	-552.8	T3R L4K K15D
160	--K---R---------D	-552.6	T3K L7R F17D
161	--K-K-K----------	-552	T3K E5K L7K
162	-R---------K----D	-551.8	A2R E12K F17D
163	--K-K--------K---	-551.7	T3K E5K L14K
164	-K------------E-D	-551	A2K K15E F17D
165	-K-K------------D	-550.9	A2K L4K F17D
166	--R-K---------D--	-550.1	T3R E5K K15D
167	-K--K-----------D	-548.2	A2K E5K F17D
168	---K--------K-E--	-548.2	L4K S13K K15E
169	------R-------E-E	-548.2	L7R K15E F17E
170	---K----------DE-	-548.1	L4K K15D S16E
171	---K--R---------E	-548.1	L4K L7R F17E
172	-R----R-------E--	-548	A2R L7R K15E
173	-R-K--R----------	-547.9	A2R L4K L7R
174	-KK-------------D	-547.6	A2K T3K F17D
175	--EK----------D--	-547.5	T3E L4K K15D
176	---K----------D-H	-547.1	L4K K15D F17H
177	---K----------DD-	-547	L4K K15D S16D
178	---K-----K----E--	-546.9	L4K A10K K15E
179	----R------K----D	-546.9	E5R E12K F17D
180	K--K----------E--	-546.2	M1K L4K K15E
181	----K-------K-E--	-545.5	E5K S13K K15E
182	----K---------DE-	-545.4	E5K K15D S16E
183	---KK-------K----	-545.4	L4K E5K S13K
184	----K-R---------E	-545.4	E5K L7R F17E
185	-R--K-R----------	-545.2	A2R E5K L7R
186	--K---------K-E--	-544.8	T3K S13K K15E
187	--K-----------DE-	-544.8	T3K K15D S16E
188	--KK--------K----	-544.8	T3K L4K S13K
189	--E-K---------D--	-544.8	T3E E5K K15D
190	--K---R---------E	-544.7	T3K L7R F17E
191	-RK---R----------	-544.6	A2R T3K L7R
192	----K---------D-H	-544.4	E5K K15D F17H
193	----K---------DD-	-544.3	E5K K15D S16D
194	----K----K----E--	-544.2	E5K A10K K15E
195	---KK----K-------	-544.2	L4K E5K A10K
196	-R---------K----E	-543.9	A2R E12K F17E
197	--K-----------D-H	-543.8	T3K K15D F17H
198	------K-------D-D	-543.7	L7K K15D F17D
199	--K-----------DD-	-543.7	T3K K15D S16D
200	--K------K----E--	-543.6	T3K A10K K15E
201	--KK-----K-------	-543.5	T3K L4K A10K
202	K---K---------E--	-543.5	M1K E5K K15E
203	K--KK------------	-543.4	M1K L4K E5K
204	-------------KD-D	-543.4	L14K K15D F17D
205	-----------KK-D--	-543.2	E12K S13K K15D
206	-K------------E-E	-543.1	A2K K15E F17E

Continued on next page

Table S3 – continued from previous page

No.	Sequence	$\Delta\tilde{E}$	Mutations
207	----R-R-------E--	-543.1	E5R L7R K15E
208	-K-K------------E	-543	A2K L4K F17E
209	---KR-R----------	-543	L4K E5R L7R
210	---K------K---D--	-542.9	L4K F11K K15D
211	K-K-----------E--	-542.9	M1K T3K K15E
212	K-KK-------------	-542.8	M1K T3K L4K
213	---K---------ED--	-542.5	L4K L14E K15D
214	--K-K-------K----	-542.1	T3K E5K S13K
215	-K----R-------D--	-542	A2K L7R K15D
216	---------K-K--D--	-541.9	A10K E12K K15D
217	K----------K--D--	-541.2	M1K E12K K15D
218	-Y-K----------D--	-541	A2Y L4K K15D
219	--K-K----K-------	-540.8	T3K E5K A10K
220	-K--K-----------E	-540.3	A2K E5K F17E
221	----K-----K---D--	-540.2	E5K F11K K15D
222	K-K-K------------	-540.1	M1K T3K E5K
223	---RK---------E--	-539.7	L4R E5K K15E
224	----K--------ED--	-539.7	E5K L14E K15D
225	-KK-------------E	-539.7	A2K T3K F17E
226	--K-R-R----------	-539.6	T3K E5R L7R
227	--K-------K---D--	-539.5	T3K F11K K15D
228	--KR----------E--	-539.1	T3K L4R K15E
229	--K----------ED--	-539.1	T3K L14E K15D
230	----R------K----E	-539	E5R E12K F17E
231	---K----------D-N	-539	L4K K15D F17N
232	---K-----R----D--	-538.9	L4K A10R K15D
233	-R--R------K-----	-538.8	A2R E5R E12K
234	-Y--K---------D--	-538.3	A2Y E5K K15D
235	-K--R---------E--	-538	A2K E5R K15E
236	-K-KR------------	-537.9	A2K L4K E5R
237	-YK-----------D--	-537.7	A2Y T3K K15D
238	---R-------K--D--	-537.4	L4R E12K K15D
239	--HK----------E--	-536.9	T3H L4K K15E
240	--KRK------------	-536.3	T3K L4R E5K
241	----K---------D-N	-536.3	E5K K15D F17N
242	----K----R----D--	-536.2	E5K A10R K15D
243	------K-------D-E	-535.8	L7K K15D F17E
244	--K-----------D-N	-535.7	T3K K15D F17N
245	--K------R----D--	-535.6	T3K A10R K15D
246	-R----K-------D--	-535.6	A2R L7K K15D
247	-------------KD-E	-535.5	L14K K15D F17E
248	-R-----------KD--	-535.3	A2R L14K K15D
249	---WK---------D--	-535.3	L4W E5K K15D
250	--KW----------D--	-534.7	T3K L4W K15D
251	---K---------DD--	-534.7	L4K L14D K15D
252	-KK-R------------	-534.6	A2K T3K E5R
253	---K---------RD--	-534.5	L4K L14R K15D
254	--H-K---------E--	-534.2	T3H E5K K15E
255	--HKK------------	-534.1	T3H L4K E5K
256	------K----K--E--	-533.9	L7K E12K K15E
257	---K--K----K-----	-533.8	L4K L7K E12K
258	------------K-D-D	-533.7	S13K K15D F17D

Continued on next page

Table S3 – continued from previous page

No.	Sequence	$\Delta\tilde{E}$	Mutations
259	-----------K-KE--	-533.6	E12K L14K K15E
260	--RK----------E--	-533.6	T3R L4K K15E
261	---K-------K-K---	-533.5	L4K E12K L14K
262	---------K----D-D	-532.4	A10K K15D F17D
263	---K----------D-Q	-532.2	L4K K15D F17Q
264	----K--------DD--	-532	E5K L14D K15D
265	--H--------K--D--	-531.9	T3H E12K K15D
266	----K--------RD--	-531.7	E5K L14R K15D
267	K-------------D-D	-531.7	M1K K15D F17D
268	------R----K----D	-531.7	L7R E12K F17D
269	---K-------M--D--	-531.4	L4K E12M K15D
270	--K----------DD--	-531.3	T3K L14D K15D
271	--K----------RD--	-531.1	T3K L14R K15D
272	----K-K----K-----	-531.1	E5K L7K E12K
273	--R-K---------E--	-530.9	T3R E5K K15E
274	----K------K-K---	-530.8	E5K E12K L14K
275	--RKK------------	-530.8	T3R L4K E5K
276	----R-K-------D--	-530.7	E5R L7K K15D
277	--K---K----K-----	-530.5	T3K L7K E12K
278	----R--------KD--	-530.4	E5R L14K K15D
279	--K--------K-K---	-530.2	T3K E12K L14K
280	--MK----------D--	-530.2	T3M L4K K15D
281	----K---------D-Q	-529.4	E5K K15D F17Q
282	-R--R-----------D	-529.4	A2R E5R F17D
283	R--K----------D--	-529	M1R L4K K15D
284	---K----------EE-	-528.9	L4K K15E S16E
285	--K-----------D-Q	-528.8	T3K K15D F17Q
286	----K------M--D--	-528.6	E5K E12M K15D
287	--R--------K--D--	-528.6	T3R E12K K15D
288	--EK----------E--	-528.2	T3E L4K K15E
289	--K--------M--D--	-528	T3K E12M K15D
290	---R----------D-D	-528	L4R K15D F17D
291	---K----------E-H	-527.9	L4K K15E F17H
292	---K----------ED-	-527.7	L4K K15E S16D
293	--M-K---------D--	-527.4	T3M E5K K15D
294	-K---------K----D	-526.6	A2K E12K F17D
295	---K----------D-S	-526.4	L4K K15D F17S
296	R---K---------D--	-526.2	M1R E5K K15D
297	----K---------EE-	-526.2	E5K K15E S16E
298	---K----------DK-	-526.1	L4K K15D S16K
299	---KK----------E-	-526.1	L4K E5K S16E
300	------------K-D-E	-525.8	S13K K15D F17E
301	-R----------K-D--	-525.6	A2R S13K K15D
302	R-K-----------D--	-525.6	M1R T3K K15D
303	--K-----------EE-	-525.5	T3K K15E S16E
304	--E-K---------E--	-525.5	T3E E5K K15E
305	--KK-----------E-	-525.5	T3K L4K S16E
306	--EKK------------	-525.4	T3E L4K E5K
307	----K---------E-H	-525.1	E5K K15E F17H
308	---KK-----------H	-525.1	L4K E5K F17H
309	---K----------D-A	-525	L4K K15D F17A
310	---K------R---D--	-525	L4K F11R K15D

Continued on next page

Table S3 – continued from previous page

No.	Sequence	$\Delta\tilde{E}$	Mutations
311	----K---------ED-	-525	E5K K15E S16D
312	---KK----------D-	-524.9	L4K E5K S16D
313	---------K----D-E	-524.6	A10K K15D F17E
314	--K-----------E-H	-524.5	T3K K15E F17H
315	--KK------------H	-524.4	T3K L4K F17H
316	------K-------E-D	-524.4	L7K K15E F17D
317	--K-----------ED-	-524.4	T3K K15E S16D
318	-R-------K----D--	-524.4	A2R A10K K15D
319	---K--K---------D	-524.3	L4K L7K F17D
320	--KK-----------D-	-524.3	T3K L4K S16D
321	-------------KE-D	-524.1	L14K K15E F17D
322	---K---------K--D	-524.1	L4K L14K F17D
323	-W-K----------D--	-524	A2W L4K K15D
324	-----------KK-E--	-523.9	E12K S13K K15E
325	-----------K--DE-	-523.9	E12K K15D S16E
326	---K-------KK----	-523.8	L4K E12K S13K
327	K-------------D-E	-523.8	M1K K15D F17E
328	------R----K----E	-523.8	L7R E12K F17E
329	----K---------D-S	-523.7	E5K K15D F17S
330	---K------E---D--	-523.6	L4K F11E K15D
331	---K------K---E--	-523.6	L4K F11K K15E
332	KR------------D--	-523.6	M1K A2R K15D
333	-R----R----K-----	-523.6	A2R L7R E12K
334	---K----------D-C	-523.6	L4K K15D F17C
335	----K---------DK-	-523.4	E5K K15D S16K
336	--E--------K--D--	-523.2	T3E E12K K15D
337	---K---------EE--	-523.2	L4K L14E K15E
338	---K----------D-G	-523.2	L4K K15D F17G
339	--K-----------D-S	-523.1	T3K K15D F17S
340	---K---K------D--	-523	L4K M8K K15D
341	-----------K--D-H	-522.8	E12K K15D F17H
342	-K----R-------E--	-522.8	A2K L7R K15E
343	--K-----------DK-	-522.8	T3K K15D S16K
344	--K-K----------E-	-522.8	T3K E5K S16E
345	-K-K--R----------	-522.7	A2K L4K L7R
346	-----------K--DD-	-522.7	E12K K15D S16D
347	---K----D-----D--	-522.7	L4K K9D K15D
348	---------K-K--E--	-522.7	A10K E12K K15E
349	---K-----K-K-----	-522.6	L4K A10K E12K
350	--H-----------D-D	-522.4	T3H K15D F17D
351	----K---------D-A	-522.3	E5K K15D F17A
352	----K-----R---D--	-522.3	E5K F11R K15D
353	---YK---------D--	-522.2	L4Y E5K K15D
354	K----------K--E--	-521.9	M1K E12K K15E
355	K--K-------K-----	-521.8	M1K L4K E12K
356	-Y-K----------E--	-521.7	A2Y L4K K15E
357	--K-K-----------H	-521.7	T3K E5K F17H
358	--K-----------D-A	-521.7	T3K K15D F17A
359	--K-------R---D--	-521.7	T3K F11R K15D
360	--KY----------D--	-521.6	T3K L4Y K15D
361	----K-K---------D	-521.6	E5K L7K F17D
362	--K-K----------D-	-521.6	T3K E5K S16D

Continued on next page

Table S3 – continued from previous page

No.	Sequence	$\Delta\tilde{E}$	Mutations
363	---K----------D-P	-521.6	L4K K15D F17P
364	-R--R-----------E	-521.5	A2R E5R F17E
365	----K--------K--D	-521.3	E5K L14K F17D
366	-W--K---------D--	-521.3	A2W E5K K15D
367	----K------KK----	-521.1	E5K E12K S13K
368	--K---K---------D	-521	T3K L7K F17D
369	----K-----E---D--	-520.9	E5K F11E K15D
370	----K-----K---E--	-520.9	E5K F11K K15E
371	---KK-----K------	-520.8	L4K E5K F11K
372	----K---------D-C	-520.8	E5K K15D F17C
373	--K----------K--D	-520.7	T3K L14K F17D
374	----R-------K-D--	-520.7	E5R S13K K15D
375	-WK-----------D--	-520.6	A2W T3K K15D
376	--K--------KK----	-520.5	T3K E12K S13K
377	----K--------EE--	-520.5	E5K L14E K15E
378	----K---------D-G	-520.5	E5K K15D F17G
379	---KK--------E---	-520.4	L4K E5K L14E
380	--NK----------D--	-520.3	T3N L4K K15D
381	----K--K------D--	-520.3	E5K M8K K15D
382	--K-------E---D--	-520.3	T3K F11E K15D
383	--K-------K---E--	-520.3	T3K F11K K15E
384	--KK------K------	-520.2	T3K L4K F11K
385	--K-----------D-C	-520.2	T3K K15D F17C
386	---K----------D-K	-520.2	L4K K15D F17K
387	---R----------D-E	-520.1	L4R K15D F17E
388	-K--K-R----------	-520	A2K E5K L7R
389	----K---D-----D--	-520	E5K K9D K15D
390	-R-R----------D--	-519.9	A2R L4R K15D
391	----K----K-K-----	-519.9	E5K A10K E12K
392	--K----------EE--	-519.9	T3K L14E K15E
393	--K-----------D-G	-519.8	T3K K15D F17G
394	--KK---------E---	-519.8	T3K L4K L14E
395	---K----------E-N	-519.7	L4K K15E F17N
396	--K----K------D--	-519.7	T3K M8K K15D
397	---K-----R----E--	-519.7	L4K A10R K15E
398	----R----K----D--	-519.5	E5R A10K K15D
399	-KK---R----------	-519.4	A2K T3K L7R
400	--K-----D-----D--	-519.4	T3K K9D K15D
401	---KK---------Q--	-519.3	L4K E5K K15Q
402	--K------K-K-----	-519.2	T3K A10K E12K
403	K---K------K-----	-519.1	M1K E5K E12K
404	--R-----------D-D	-519.1	T3R K15D F17D
405	-Y--K---------E--	-519	A2Y E5K K15E
406	-Y-KK------------	-518.9	A2Y L4K E5K
407	----K---------D-P	-518.8	E5K K15D F17P
408	-K---------K----E	-518.7	A2K E12K F17E
409	K---R---------D--	-518.7	M1K E5R K15D
410	--KK----------Q--	-518.7	T3K L4K K15Q
411	----R-R----K-----	-518.7	E5R L7R E12K
412	----------KK--D--	-518.6	F11K E12K K15D
413	K-K--------K-----	-518.5	M1K T3K E12K
414	-YK-----------E--	-518.4	A2Y T3K K15E

Continued on next page

Table S3 – continued from previous page

No.	Sequence	$\Delta\tilde{E}$	Mutations
415	-YKK-------------	-518.3	A2Y T3K L4K
416	--K-----------D-P	-518.2	T3K K15D F17P
417	---R-------K--E--	-518.2	L4R E12K K15E
418	-----------K-ED--	-518.2	E12K L14E K15D
419	--N-K---------D--	-517.6	T3N E5K K15D
420	--YK----------D--	-517.6	T3Y L4K K15D
421	--K-K-----K------	-517.5	T3K E5K F11K
422	----K---------D-K	-517.5	E5K K15D F17K
423	--K-K--------E---	-517.1	T3K E5K L14E
424	----K---------E-N	-517	E5K K15E F17N
425	----K----R----E--	-517	E5K A10R K15E
426	---KK-----------N	-516.9	L4K E5K F17N
427	---KK----R-------	-516.9	L4K E5K A10R
428	--K-----------D-K	-516.8	T3K K15D F17K
429	-Y---------K--D--	-516.7	A2Y E12K K15D
430	------K-------E-E	-516.5	L7K K15E F17E
431	---K--K---------E	-516.4	L4K L7K F17E
432	--K-----------E-N	-516.4	T3K K15E F17N
433	---KK---------G--	-516.4	L4K E5K K15G
434	--K------R----E--	-516.3	T3K A10R K15E
435	-R----K-------E--	-516.3	A2R L7K K15E
436	--KK------------N	-516.3	T3K L4K F17N
437	--KK-----R-------	-516.3	T3K L4K A10R
438	-R-K--K----------	-516.3	A2R L4K L7K
439	-------------KE-E	-516.2	L14K K15E F17E
440	---K---------K--E	-516.2	L4K L14K F17E
441	-R-----------KE--	-516.1	A2R L14K K15E
442	---WK---------E--	-516	L4W E5K K15E
443	-R-K---------K---	-516	A2R L4K L14K
444	--K-K---------Q--	-516	T3K E5K K15Q
445	--KK----------G--	-515.8	T3K L4K K15G
446	-YK-K------------	-515.6	A2Y T3K E5K
447	--KW----------E--	-515.4	T3K L4W K15E
448	---K---------DE--	-515.4	L4K L14D K15E
449	---RK------K-----	-515.4	L4R E5K E12K
450	---K--------R-D--	-515.3	L4K S13R K15D
451	---K---------RE--	-515.2	L4K L14R K15E
452	------R------KD--	-515.2	L7R L14K K15D
453	---RR---------D--	-515	L4R E5R K15D
454	--Y-K---------D--	-514.8	T3Y E5K K15D
455	--KR-------K-----	-514.8	T3K L4R E12K
456	-----------K--D-N	-514.7	E12K K15D F17N
457	---------R-K--D--	-514.6	A10R E12K K15D
458	--H-----------D-E	-514.6	T3H K15D F17E
459	------------K-E-D	-514.4	S13K K15E F17D
460	--------------DED	-514.4	K15D S16E F17D
461	-RH-----------D--	-514.4	A2R T3H K15D
462	---K--------K---D	-514.4	L4K S13K F17D
463	-R----R---------D	-514.1	A2R L7R F17D
464	---W-------K--D--	-513.7	L4W E12K K15D
465	--E-----------D-D	-513.7	T3E K15D F17D
466	----K-K---------E	-513.7	E5K L7K F17E

Continued on next page

Table S3 – continued from previous page

No.	Sequence	$\Delta\tilde{E}$	Mutations
467	-K--R------K-----	-513.7	A2K E5R E12K
468	--K-K-----------N	-513.6	T3K E5K F17N
469	--K-K----R-------	-513.5	T3K E5K A10R
470	-R--K-K----------	-513.5	A2R E5K L7K
471	---KN---------D--	-513.5	L4K E5N K15D
472	----K--------K--E	-513.4	E5K L14K F17E
473	-R--K--------K---	-513.3	A2R E5K L14K
474	--------------DDD	-513.2	K15D S16D F17D
475	---------K----E-D	-513.2	A10K K15E F17D
476	---K-----K------D	-513.1	L4K A10K F17D
477	--K---K---------E	-513.1	T3K L7K F17E
478	--K-K---------G--	-513	T3K E5K K15G
479	---KK---------Y--	-513	L4K E5K K15Y
480	-RK---K----------	-512.9	A2R T3K L7K
481	---K----------E-Q	-512.9	L4K K15E F17Q
482	--K----------K--E	-512.8	T3K L14K F17E
483	----K--------DE--	-512.7	E5K L14D K15E
484	-RK----------K---	-512.7	A2R T3K L14K
485	--H--------K--E--	-512.6	T3H E12K K15E
486	--KWK------------	-512.6	T3K L4W E5K
487	---KK--------D---	-512.6	L4K E5K L14D
488	----K-------R-D--	-512.6	E5K S13R K15D
489	---KK---------A--	-512.6	L4K E5K K15A
490	--HK-------K-----	-512.6	T3H L4K E12K
491	----K--------RE--	-512.5	E5K L14R K15E
492	K-------------E-D	-512.4	M1K K15E F17D
493	---KK--------R---	-512.4	L4K E5K L14R
494	K--K------------D	-512.4	M1K L4K F17D
495	--KK----------Y--	-512.3	T3K L4K K15Y
496	--WK----------D--	-512.2	T3W L4K K15D
497	---K-------M--E--	-512.1	L4K E12M K15E
498	--K----------DE--	-512.1	T3K L14D K15E
499	--KK---------D---	-512	T3K L4K L14D
500	--K---------R-D--	-512	T3K S13R K15D
501	--KK----------A--	-512	T3K L4K K15A
502	---KK---------T--	-511.9	L4K E5K K15T
503	--K----------RE--	-511.9	T3K L14R K15E
504	--KK---------R---	-511.8	T3K L4K L14R
505	---K------D---D--	-511.7	L4K F11D K15D
506	----K-------K---D	-511.6	E5K S13K F17D
507	----R-K-------E--	-511.4	E5R L7K K15E
508	---KR-K----------	-511.4	L4K E5R L7K
509	--KK----------T--	-511.2	T3K L4K K15T
510	--R-----------D-E	-511.2	T3R K15D F17E
511	----R--------KE--	-511.2	E5R L14K K15E
512	---KR--------K---	-511.1	L4K E5R L14K
513	-RR-----------D--	-511	A2R T3R K15D
514	--K---------K---D	-511	T3K S13K F17D
515	--MK----------E--	-510.9	T3M L4K K15E
516	-K----K-------D--	-510.4	A2K L7K K15D
517	-----------K-DD--	-510.4	E12K L14D K15D
518	----K----K------D	-510.4	E5K A10K F17D

Continued on next page

Table S3 – continued from previous page

No.	Sequence	$\Delta\tilde{E}$	Mutations
519	---K----R-----D--	-510.4	L4K K9R K15D
520	--K-N---------D--	-510.2	T3K E5N K15D
521	----K---------E-Q	-510.2	E5K K15E F17Q
522	-----------K-RD--	-510.2	E12K L14R K15D
523	-K-----------KD--	-510.1	A2K L14K K15D
524	---KK-----------Q	-510.1	L4K E5K F17Q
525	---K---R------D--	-510	L4K M8R K15D
526	--H-K------K-----	-509.8	T3H E5K E12K
527	--K------K------D	-509.8	T3K A10K F17D
528	R--K----------E--	-509.7	M1R L4K K15E
529	---KK---------H--	-509.7	L4K E5K K15H
530	K---K-----------D	-509.6	M1K E5K F17D
531	--K-K---------Y--	-509.6	T3K E5K K15Y
532	--K-----------E-Q	-509.6	T3K K15E F17Q
533	--W-K---------D--	-509.5	T3W E5K K15D
534	--KK------------Q	-509.5	T3K L4K F17Q
535	--H-R---------D--	-509.5	T3H E5R K15D
536	----K------M--E--	-509.4	E5K E12M K15E
537	---KK------M-----	-509.3	L4K E5K E12M
538	--R--------K--E--	-509.3	T3R E12K K15E
539	--K-K--------D---	-509.3	T3K E5K L14D
540	--K-K---------A--	-509.3	T3K E5K K15A
541	----R-R---------D	-509.2	E5R L7R F17D
542	--RK-------K-----	-509.2	T3R L4K E12K
543	----------K---D-D	-509.1	F11K K15D F17D
544	--KK----------H--	-509.1	T3K L4K K15H
545	--K-K--------R---	-509.1	T3K E5K L14R
546	K-K-------------D	-509	M1K T3K F17D
547	----K-----D---D--	-509	E5K F11D K15D
548	---KK---------S--	-508.8	L4K E5K K15S
549	--K--------M--E--	-508.8	T3K E12M K15E
550	---R----------E-D	-508.7	L4R K15E F17D
551	--KK-------M-----	-508.7	T3K L4K E12M
552	-------------ED-D	-508.7	L14E K15D F17D
553	--K-K---------T--	-508.5	T3K E5K K15T
554	--K-------D---D--	-508.4	T3K F11D K15D
555	--KK----------S--	-508.2	T3K L4K K15S
556	--M-K---------E--	-508.2	T3M E5K K15E
557	--MKK------------	-508.1	T3M L4K E5K
558	--K-R-K----------	-508	T3K E5R L7K
559	-----------K--D-Q	-507.9	E12K K15D F17Q
560	--K-R--------K---	-507.7	T3K E5R L14K
561	----K---R-----D--	-507.7	E5K K9R K15D
562	----K--R------D--	-507.3	E5K M8R K15D
563	-Y------------D-D	-507.2	A2Y K15D F17D
564	---K----------E-S	-507.2	L4K K15E F17S
565	--K-----R-----D--	-507	T3K K9R K15D
566	R---K---------E--	-507	M1R E5K K15E
567	R--KK------------	-506.9	M1R L4K E5K
568	---K----------EK-	-506.9	L4K K15E S16K
569	--K-K-----------Q	-506.8	T3K E5K F17Q
570	--K----R------D--	-506.7	T3K M8R K15D

Continued on next page

Table S3 – continued from previous page

No.	Sequence	$\Delta\tilde{E}$	Mutations
571	------------K-E-E	-506.5	S13K K15E F17E
572	--------------DEE	-506.5	K15D S16E F17E
573	--R-K------K-----	-506.5	T3R E5K E12K
574	---K--------K---E	-506.5	L4K S13K F17E
575	-R----------K-E--	-506.4	A2R S13K K15E
576	R-K-----------E--	-506.4	M1R T3K K15E
577	--K-K---------H--	-506.3	T3K E5K K15H
578	-R------------DE-	-506.3	A2R K15D S16E
579	-R-K--------K----	-506.3	A2R L4K S13K
580	R-KK-------------	-506.3	M1R T3K L4K
581	-R----R---------E	-506.3	A2R L7R F17E
582	--R-R---------D--	-506.1	T3R E5R K15D
583	--K-K------M-----	-506	T3K E5K E12M
584	---RK-----------D	-505.9	L4R E5K F17D
585	--M--------K--D--	-505.9	T3M E12K K15D
586	--E-----------D-E	-505.9	T3E K15D F17E
587	---K----------E-A	-505.8	L4K K15E F17A
588	---K------R---E--	-505.8	L4K F11R K15E
589	-RE-----------D--	-505.7	A2R T3E K15D
590	------R-----K-D--	-505.5	L7R S13K K15D
591	--K-K---------S--	-505.5	T3K E5K K15S
592	--------------DDE	-505.4	K15D S16D F17E
593	---KK---------L--	-505.3	L4K E5K K15L
594	---------K----E-E	-505.3	A10K K15E F17E
595	-R------------D-H	-505.3	A2R K15D F17H
596	--KR------------D	-505.3	T3K L4R F17D
597	---K-----K------E	-505.2	L4K A10K F17E
598	---------R----D-D	-505.2	A10R K15D F17D
599	-R------------DD-	-505.2	A2R K15D S16D
600	-R-------K----E--	-505.1	A2R A10K K15E
601	-R-K-----K-------	-505.1	A2R L4K A10K
602	---K----E-----D--	-505	L4K K9E K15D
603	---K---E------D--	-504.8	L4K M8E K15D
604	--KK----------L--	-504.7	T3K L4K K15L
605	-W-K----------E--	-504.7	A2W L4K K15E
606	R----------K--D--	-504.7	M1R E12K K15D
607	---KQ---------D--	-504.6	L4K E5Q K15D
608	-----------K--EE-	-504.6	E12K K15E S16E
609	K-------------E-E	-504.6	M1K K15E F17E
610	---K-------K---E-	-504.5	L4K E12K S16E
611	K--K------------E	-504.5	M1K L4K F17E
612	----K---------E-S	-504.4	E5K K15E F17S
613	---K------E---E--	-504.4	L4K F11E K15E
614	KR------------E--	-504.4	M1K A2R K15E
615	---KK-----------S	-504.4	L4K E5K F17S
616	KR-K-------------	-504.3	M1K A2R L4K
617	---K----------E-C	-504.3	L4K K15E F17C
618	---W----------D-D	-504.3	L4W K15D F17D
619	------R--K----D--	-504.2	L7R A10K K15D
620	-K--R-----------D	-504.2	A2K E5R F17D
621	----K---------EK-	-504.1	E5K K15E S16K
622	---KK---------P--	-504.1	L4K E5K K15P

Continued on next page

Table S3 – continued from previous page

No.	Sequence	$\Delta\tilde{E}$	Mutations
623	---KK----------K-	-504.1	L4K E5K S16K
624	--E--------K--E--	-503.9	T3E E12K K15E
625	---K----------E-G	-503.9	L4K K15E F17G
626	--EK-------K-----	-503.9	T3E L4K E12K
627	--K-----------E-S	-503.8	T3K K15E F17S
628	---K---K------E--	-503.8	L4K M8K K15E
629	--KK------------S	-503.8	T3K L4K F17S
630	----K-------K---E	-503.8	E5K S13K F17E
631	--IK----------D--	-503.6	T3I L4K K15D
632	-R--K-------K----	-503.6	A2R E5K S13K
633	-----------K--E-H	-503.6	E12K K15E F17H
634	R-K-K------------	-503.6	M1R T3K E5K
635	--K-----------EK-	-503.5	T3K K15E S16K
636	K-----R-------D--	-503.5	M1K L7R K15D
637	---K-------K----H	-503.5	L4K E12K F17H
638	--KK----------P--	-503.5	T3K L4K K15P
639	-----------K--ED-	-503.5	E12K K15E S16D
640	--KK-----------K-	-503.4	T3K L4K S16K
641	---K----D-----E--	-503.4	L4K K9D K15E
642	---K-------K---D-	-503.4	L4K E12K S16D
643	--H-----------E-D	-503.2	T3H K15E F17D
644	--K---------K---E	-503.1	T3K S13K F17E
645	--HK------------D	-503.1	T3H L4K F17D
646	----K---------E-A	-503.1	E5K K15E F17A
647	----K-----R---E--	-503	E5K F11R K15E
648	---KK-----------A	-503	L4K E5K F17A
649	---YK---------E--	-503	L4Y E5K K15E
650	---KK-----R------	-503	L4K E5K F11R
651	-RK---------K----	-503	A2R T3K S13K
652	----K----K------E	-502.5	E5K A10K F17E
653	--K-----------E-A	-502.4	T3K K15E F17A
654	--K-------R---E--	-502.4	T3K F11R K15E
655	--KK------------A	-502.4	T3K L4K F17A
656	--KY----------E--	-502.4	T3K L4Y K15E
657	--KK------R------	-502.3	T3K L4K F11R
658	-R--K----K-------	-502.3	A2R E5K A10K
659	---K----------E-P	-502.3	L4K K15E F17P
660	----K---E-----D--	-502.3	E5K K9E K15D
661	-----------K--D-S	-502.1	E12K K15D F17S
662	----K--E------D--	-502	E5K M8E K15D
663	--K-K---------L--	-502	T3K E5K K15L
664	-W--K---------E--	-502	A2W E5K K15E
665	-W-KK------------	-501.9	A2W L4K E5K
666	--K------K------E	-501.9	T3K A10K F17E
667	-----------K--DK-	-501.8	E12K K15D S16K
668	----K------K---E-	-501.8	E5K E12K S16E
669	K---K-----------E	-501.8	M1K E5K F17E
670	-RK------K-------	-501.7	A2R T3K A10K
671	--K-----E-----D--	-501.7	T3K K9E K15D
672	----K-----E---E--	-501.7	E5K F11E K15E
673	---KK-----E------	-501.6	L4K E5K F11E
674	KR--K------------	-501.6	M1K A2R E5K

Continued on next page

Table S3 – continued from previous page

No.	Sequence	$\Delta\tilde{E}$	Mutations
675	----K---------E-C	-501.6	E5K K15E F17C
676	---KK-----------C	-501.5	L4K E5K F17C
677	----R-------K-E--	-501.5	E5R S13K K15E
678	--K----E------D--	-501.4	T3K M8E K15D
679	----R---------DE-	-501.4	E5R K15D S16E
680	---K----------D-M	-501.4	L4K K15D F17M
681	---KR-------K----	-501.4	L4K E5R S13K
682	-WK-----------E--	-501.4	A2W T3K K15E
683	----R-R---------E	-501.3	E5R L7R F17E
684	-WKK-------------	-501.3	A2W T3K L4K
685	--K-Q---------D--	-501.3	T3K E5Q K15D
686	----------K---D-E	-501.3	F11K K15D F17E
687	----K---------E-G	-501.2	E5K K15E F17G
688	--K--------K---E-	-501.2	T3K E12K S16E
689	-R--R-R----------	-501.2	A2R E5R L7R
690	--E-K------K-----	-501.2	T3E E5K E12K
691	K-K-------------E	-501.1	M1K T3K F17E
692	---KK-----------G	-501.1	L4K E5K F17G
693	--NK----------E--	-501.1	T3N L4K K15E
694	-R--------K---D--	-501.1	A2R F11K K15D
695	----K--K------E--	-501	E5K M8K K15E
696	--K-------E---E--	-501	T3K F11E K15E
697	--K-K-----------S	-501	T3K E5K F17S
698	---KK--K---------	-501	L4K E5K M8K
699	--KK------E------	-501	T3K L4K F11E
700	KRK--------------	-501	M1K A2R T3K
701	--K-----------E-C	-501	T3K K15E F17C
702	-------------DD-D	-500.9	L14D K15D F17D
703	---K----------E-K	-500.9	L4K K15E F17K
704	--KK------------C	-500.9	T3K L4K F17C
705	--I-K---------D--	-500.9	T3I E5K K15D
706	---R----------E-E	-500.8	L4R K15E F17E
707	-------------ED-E	-500.8	L14E K15D F17E
708	----K------K----H	-500.8	E5K E12K F17H
709	---K--------D-D--	-500.8	L4K S13D K15D
710	--E-R---------D--	-500.8	T3E E5R K15D
711	-----------K--D-A	-500.8	E12K K15D F17A
712	--K-K---------P--	-500.7	T3K E5K K15P
713	----------RK--D--	-500.7	F11R E12K K15D
714	--K-K----------K-	-500.7	T3K E5K S16K
715	----K---D-----E--	-500.7	E5K K9D K15E
716	-------------RD-D	-500.7	L14R K15D F17D
717	---Y-------K--D--	-500.7	L4Y E12K K15D
718	----K------K---D-	-500.7	E5K E12K S16D
719	---KK---D--------	-500.6	L4K E5K K9D
720	-R-R----------E--	-500.6	A2R L4R K15E
721	-R-----------ED--	-500.6	A2R L14E K15D
722	--FK----------D--	-500.6	T3F L4K K15D
723	--K-----------E-G	-500.6	T3K K15E F17G
724	--KK------------G	-500.5	T3K L4K F17G
725	-K----------K-D--	-500.4	A2K S13K K15D
726	--K----K------E--	-500.4	T3K M8K K15E

Continued on next page

Table S3 – continued from previous page

No.	Sequence	$\Delta\tilde{E}$	Mutations
727	--H-K-----------D	-500.4	T3H E5K F17D
728	----R---------D-H	-500.4	E5R K15D F17H
729	--KK---K---------	-500.4	T3K L4K M8K
730	----R---------DD-	-500.3	E5R K15D S16D
731	----R----K----E--	-500.2	E5R A10K K15E
732	--K--------K----H	-500.2	T3K E12K F17H
733	---KR----K-------	-500.1	L4K E5R A10K
734	--K-----D-----E--	-500.1	T3K K9D K15E
735	------K----K----D	-500	L7K E12K F17D
736	--K--------K---D-	-500	T3K E12K S16D
737	--KK----D--------	-500	T3K L4K K9D
738	---KC---------D--	-500	L4K E5C K15D
739	--R-----------E-D	-499.8	T3R K15E F17D
740	-----------K-K--D	-499.8	E12K L14K F17D
741	---R--R-------D--	-499.8	L4R L7R K15D
742	--RK------------D	-499.8	T3R L4K F17D
743	-W---------K--D--	-499.7	A2W E12K K15D
744	---KS---------D--	-499.7	L4K E5S K15D
745	--K-K-----------A	-499.6	T3K E5K F17A
746	--K-K-----R------	-499.6	T3K E5K F11R
747	---KW---------D--	-499.6	L4K E5W K15D
748	----K---------E-P	-499.6	E5K K15E F17P
749	--KYK------------	-499.6	T3K L4Y E5K
750	---KK-----------P	-499.5	L4K E5K F17P
751	K---R---------E--	-499.5	M1K E5R K15E
752	K--KR------------	-499.4	M1K L4K E5R
753	-Y------------D-E	-499.4	A2Y K15D F17E
754	----------EK--D--	-499.4	F11E E12K K15D
755	----------KK--E--	-499.3	F11K E12K K15E
756	---K------KK-----	-499.3	L4K F11K E12K
757	-----------K--D-C	-499.3	E12K K15D F17C
758	-K-------K----D--	-499.2	A2K A10K K15D
759	--K-----------E-P	-499	T3K K15E F17P
760	-----------K-EE--	-498.9	E12K L14E K15E
761	--KK------------P	-498.9	T3K L4K F17P
762	-----------K--D-G	-498.9	E12K K15D F17G
763	---K-------K-E---	-498.8	L4K E12K L14E
764	---KG---------D--	-498.8	L4K E5G K15D
765	-------K---K--D--	-498.7	M8K E12K K15D
766	----K---------D-M	-498.7	E5K K15D F17M
767	-WK-K------------	-498.6	A2W T3K E5K
768	KK------------D--	-498.5	M1K A2K K15D
769	-K----R----K-----	-498.4	A2K L7R E12K
770	--------D--K--D--	-498.4	K9D E12K K15D
771	--N-K---------E--	-498.4	T3N E5K K15E
772	--YK----------E--	-498.3	T3Y L4K K15E
773	--NKK------------	-498.3	T3N L4K E5K
774	--K-K-----E------	-498.2	T3K E5K F11E
775	----K---------E-K	-498.2	E5K K15E F17K
776	--K-K-----------C	-498.2	T3K E5K F17C
777	---KK-----------K	-498.1	L4K E5K F17K
778	--K-----------D-M	-498.1	T3K K15D F17M

Continued on next page

Table S3 – continued from previous page

No.	Sequence	$\Delta\tilde{E}$	Mutations
779	--K-R-------K----	-498	T3K E5R S13K
780	----K-------D-D--	-498	E5K S13D K15D
781	---RK-----------E	-498	L4R E5K F17E
782	--LK----------D--	-498	T3L L4K K15D
783	--F-K---------D--	-497.9	T3F E5K K15D
784	-R-RK------------	-497.8	A2R L4R E5K
785	--K-K-----------G	-497.8	T3K E5K F17G
786	---K-------K--Q--	-497.8	L4K E12K K15Q
787	---K-D--------D--	-497.7	L4K K6D K15D
788	---KK---------C--	-497.7	L4K E5K K15C
789	--K-K--K---------	-497.6	T3K E5K M8K
790	-----------M--D-D	-497.6	E12M K15D F17D
791	--K-----------E-K	-497.6	T3K K15E F17K
792	--KK------------K	-497.5	T3K L4K F17K
793	-Y---------K--E--	-497.4	A2Y E12K K15E
794	--K---------D-D--	-497.4	T3K S13D K15D
795	--KR------------E	-497.4	T3K L4R F17E
796	-Y-K-------K-----	-497.4	A2Y L4K E12K
797	---------R----D-E	-497.3	A10R K15D F17E
798	--K-K---D--------	-497.3	T3K E5K K9D
799	-----------K--D-P	-497.3	E12K K15D F17P
800	-RKR-------------	-497.2	A2R T3K L4R
801	-R------------D-N	-497.2	A2R K15D F17N
802	-R-------R----D--	-497.1	A2R A10R K15D
803	--KK----------C--	-497	T3K L4K K15C
804	--R-K-----------D	-497	T3R E5K F17D
805	---K----------D-I	-496.8	L4K K15D F17I
806	--K-R----K-------	-496.8	T3K E5R A10K
807	---K-R--------D--	-496.7	L4K K6R K15D
808	--K-C---------D--	-496.6	T3K E5C K15D
809	----K-----KK-----	-496.5	E5K F11K E12K
810	--M-----------D-D	-496.4	T3M K15D F17D
811	---W----------D-E	-496.4	L4W K15D F17E
812	--K-S---------D--	-496.3	T3K E5S K15D
813	-K--R-----------E	-496.3	A2K E5R F17E
814	--K-W---------D--	-496.3	T3K E5W K15D
815	-R-W----------D--	-496.2	A2R L4W K15D
816	--K-K-----------P	-496.2	T3K E5K F17P
817	----R-----K---D--	-496.2	E5R F11K K15D
818	----K------K-E---	-496.1	E5K E12K L14E
819	---K--------R-E--	-496.1	L4K S13R K15E
820	--N--------K--D--	-496.1	T3N E12K K15D
821	K-K-R------------	-496	M1K T3K E5R
822	--K-------KK-----	-495.9	T3K F11K E12K
823	------R------KE--	-495.9	L7R L14K K15E
824	-----------K--D-K	-495.9	E12K K15D F17K
825	---K--R------K---	-495.9	L4K L7R L14K
826	---RR---------E--	-495.7	L4R E5R K15E
827	----R--------ED--	-495.7	E5R L14E K15D
828	---K----------DF-	-495.7	L4K K15D S16F
829	---K-------R--D--	-495.7	L4K E12R K15D
830	---KA---------D--	-495.7	L4K E5A K15D

Continued on next page

Table S3 – continued from previous page

No.	Sequence	$\Delta\tilde{E}$	Mutations
831	--Y-K---------E--	-495.6	T3Y E5K K15E
832	--YKK------------	-495.5	T3Y L4K E5K
833	--K--------K-E---	-495.5	T3K E12K L14E
834	---KM---------D--	-495.5	L4K E5M K15D
835	-----------K--E-N	-495.4	E12K K15E F17N
836	--K-G---------D--	-495.4	T3K E5G K15D
837	---------R-K--E--	-495.4	A10R E12K K15E
838	---K-------K----N	-495.4	L4K E12K F17N
839	---K-----R-K-----	-495.3	L4K A10R E12K
840	--H-----------E-E	-495.3	T3H K15E F17E
841	--L-K---------D--	-495.3	T3L E5K K15D
842	--HK------------E	-495.2	T3H L4K F17E
843	R-------------D-D	-495.2	M1R K15D F17D
844	--------------EED	-495.1	K15E S16E F17D
845	-RH-----------E--	-495.1	A2R T3H K15E
846	---K-----------ED	-495.1	L4K S16E F17D
847	----K------K--Q--	-495	E5K E12K K15Q
848	-RHK-------------	-495	A2R T3H L4K
849	----KD--------D--	-494.9	E5K K6D K15D
850	---K----------D-V	-494.9	L4K K15D F17V
851	---K-------K--G--	-494.8	L4K E12K K15G
852	--K-K-----------K	-494.8	T3K E5K F17K
853	-K-R----------D--	-494.7	A2K L4R K15D
854	-Y--K------K-----	-494.6	A2Y E5K E12K
855	---W-------K--E--	-494.5	L4W E12K K15E
856	--E-----------E-D	-494.5	T3E K15E F17D
857	--K--------K--Q--	-494.4	T3K E12K K15Q
858	--EK------------D	-494.4	T3E L4K F17D
859	--K--D--------D--	-494.3	T3K K6D K15D
860	--K-K---------C--	-494.3	T3K E5K K15C
861	---KN---------E--	-494.3	L4K E5N K15E
862	-Y--R---------D--	-494.3	A2Y E5R K15D
863	--H---R-------D--	-494.2	T3H L7R K15D
864	----K---------D-I	-494.1	E5K K15D F17I
865	-YK--------K-----	-494	A2Y T3K E12K
866	--------------EDD	-494	K15E S16D F17D
867	----KR--------D--	-493.9	E5K K6R K15D
868	---K-----------DD	-493.9	L4K S16D F17D
869	---K-------W--D--	-493.9	L4K E12W K15D
870	---KK---------V--	-493.8	L4K E5K K15V
871	--K-----------D-I	-493.5	T3K K15D F17I
872	----K-------R-E--	-493.4	E5K S13R K15E
873	--K--R--------D--	-493.3	T3K K6R K15D
874	---KK-------R----	-493.3	L4K E5K S13R
875	--Y--------K--D--	-493.3	T3Y E12K K15D
876	--KK----------V--	-493.2	T3K L4K K15V
877	---KF---------D--	-493.2	L4K E5F K15D
878	---KK---------R--	-493.2	L4K E5K K15R
879	----K-R------K---	-493.1	E5K L7R L14K
880	-------------DD-E	-493	L14D K15D F17E
881	----K---------DF-	-493	E5K K15D S16F
882	----K------R--D--	-493	E5K E12R K15D

Continued on next page

Table S3 – continued from previous page

No.	Sequence	$\Delta\tilde{E}$	Mutations
883	--WK----------E--	-493	T3W L4K K15E
884	-R-----------DD--	-492.9	A2R L14D K15D
885	-------------RD-E	-492.8	L14R K15D F17E
886	--K---------R-E--	-492.7	T3K S13R K15E
887	---K---D------D--	-492.7	L4K M8D K15D
888	--KK--------R----	-492.7	T3K L4K S13R
889	----K------K----N	-492.7	E5K E12K F17N
890	-R-----------RD--	-492.6	A2R L14R K15D
891	----K----R-K-----	-492.6	E5K A10R E12K
892	--KK----------R--	-492.6	T3K L4K K15R
893	--K---R------K---	-492.5	T3K L7R L14K
894	--H-K-----------E	-492.5	T3H E5K F17E
895	---K------D---E--	-492.5	L4K F11D K15E
896	--K-----------DF-	-492.4	T3K K15D S16F
897	--K--------R--D--	-492.4	T3K E12R K15D
898	--------------DKD	-492.4	K15D S16K F17D
899	--K-A---------D--	-492.4	T3K E5A K15D
900	----K----------ED	-492.3	E5K S16E F17D
901	-RH-K------------	-492.3	A2R T3H E5K
902	--KRR------------	-492.3	T3K L4R E5R
903	----R---------D-N	-492.3	E5R K15D F17N
904	----R----R----D--	-492.2	E5R A10R K15D
905	----K---------D-V	-492.2	E5K K15D F17V
906	------K----K----E	-492.2	L7K E12K F17E
907	--K-M---------D--	-492.1	T3K E5M K15D
908	----K------K--G--	-492.1	E5K E12K K15G
909	--K--------K----N	-492	T3K E12K F17N
910	--K------R-K-----	-492	T3K A10R E12K
911	-R----K----K-----	-492	A2R L7K E12K
912	--R-----------E-E	-491.9	T3R K15E F17E
913	-----------K-K--E	-491.9	E12K L14K F17E
914	--RK------------E	-491.9	T3R L4K F17E
915	-RR-----------E--	-491.8	A2R T3R K15E
916	--K------------ED	-491.7	T3K S16E F17D
917	-R---------K-K---	-491.7	A2R E12K L14K
918	-RRK-------------	-491.7	A2R T3R L4K
919	---WK------K-----	-491.7	L4W E5K E12K
920	--E-K-----------D	-491.7	T3E E5K F17D
921	--K-----------D-V	-491.6	T3K K15D F17V
922	--K--------K--G--	-491.5	T3K E12K K15G
923	---K-------K--Y--	-491.4	L4K E12K K15Y
924	---WR---------D--	-491.3	L4W E5R K15D
925	----------R---D-D	-491.3	F11R K15D F17D
926	---Y----------D-D	-491.2	L4Y K15D F17D
927	----K----------DD	-491.2	E5K S16D F17D
928	----K------W--D--	-491.2	E5K E12W K15D
929	-K----K-------E--	-491.2	A2K L7K K15E
930	-----------K-DE--	-491.1	E12K L14D K15E
931	---K----R-----E--	-491.1	L4K K9R K15E
932	-K-K--K----------	-491.1	A2K L4K L7K
933	--KW-------K-----	-491.1	T3K L4W E12K
934	---K-------K-D---	-491.1	L4K E12K L14D

Continued on next page

Table S3 – continued from previous page

No.	Sequence	$\Delta\tilde{E}$	Mutations
935	-----------KR-D--	-491	E12K S13R K15D
936	---K-------K--A--	-491	L4K E12K K15A
937	--K-N---------E--	-490.9	T3K E5N K15E
938	-----------K-RE--	-490.9	E12K L14R K15E
939	--R---R-------D--	-490.9	T3R L7R K15D
940	-K-----------KE--	-490.9	A2K L14K K15E
941	--KKN------------	-490.9	T3K L4K E5N
942	---K-------K-R---	-490.8	L4K E12K L14R
943	-K-K---------K---	-490.8	A2K L4K L14K
944	---K---R------E--	-490.8	L4K M8R K15E
945	---K-----E----D--	-490.7	L4K A10E K15D
946	--K------------DD	-490.6	T3K S16D F17D
947	--K--------W--D--	-490.6	T3K E12W K15D
948	--K-K---------V--	-490.5	T3K E5K K15V
949	-R------------D-Q	-490.3	A2R K15D F17Q
950	---K-------K--T--	-490.3	L4K E12K K15T
951	--W-K---------E--	-490.3	T3W E5K K15E
952	-W------------D-D	-490.2	A2W K15D F17D
953	--H-R---------E--	-490.2	T3H E5R K15E
954	--WKK------------	-490.2	T3W L4K E5K
955	---KT---------D--	-490.2	L4K E5T K15D
956	--HKR------------	-490.1	T3H L4K E5R
957	-----------KK---D	-490.1	E12K S13K F17D
958	----K--D------D--	-490	E5K M8D K15D
959	--K-K-------R----	-489.9	T3K E5K S13R
960	----------E---D-D	-489.9	F11E K15D F17D
961	---K----------D-R	-489.9	L4K K15D F17R
962	----------K---E-D	-489.9	F11K K15E F17D
963	--K-F---------D--	-489.8	T3K E5F K15D
964	--K-K---------R--	-489.8	T3K E5K K15R
965	---K------K-----D	-489.8	L4K F11K F17D
966	----K-----D---E--	-489.7	E5K F11D K15E
967	-----------M--D-E	-489.7	E12M K15D F17E
968	---KK-----D------	-489.7	L4K E5K F11D
969	-R---------M--D--	-489.5	A2R E12M K15D
970	-------------EE-D	-489.4	L14E K15E F17D
971	---K---------E--D	-489.4	L4K L14E F17D
972	--K----D------D--	-489.4	T3K M8D K15D
973	-------K------D-D	-489.3	M8K K15D F17D
974	----N------K--D--	-489.2	E5N E12K K15D
975	-KH-----------D--	-489.2	A2K T3H K15D
976	--R-K-----------E	-489.1	T3R E5K F17E
977	--K-------D---E--	-489.1	T3K F11D K15E
978	--KK------D------	-489	T3K L4K F11D
979	-RR-K------------	-489	A2R T3R E5K
980	-K----R---------D	-489	A2K L7R F17D
981	--------D-----D-D	-488.9	K9D K15D F17D
982	---------K-K----D	-488.8	A10K E12K F17D
983	----K------K--Y--	-488.7	E5K E12K K15Y
984	-----------K--E-Q	-488.6	E12K K15E F17Q
985	---K-------K----Q	-488.6	L4K E12K F17Q
986	--M-----------D-E	-488.5	T3M K15D F17E

Continued on next page

Table S3 – continued from previous page

No.	Sequence	$\Delta\tilde{E}$	Mutations
987	----K---R-----E--	-488.4	E5K K9R K15E
988	-K--K-K----------	-488.4	A2K E5K L7K
989	-RM-----------D--	-488.4	A2R T3M K15D
990	----K------K-D---	-488.3	E5K E12K L14D
991	---KK---R--------	-488.3	L4K E5K K9R
992	----K------K--A--	-488.3	E5K E12K K15A
993	---K----------Q-D	-488.3	L4K K15Q F17D
994	---K-------K--H--	-488.1	L4K E12K K15H
995	----K------K-R---	-488.1	E5K E12K L14R
996	-K--K--------K---	-488.1	A2K E5K L14K
997	K----------K----D	-488.1	M1K E12K F17D
998	--K--------K--Y--	-488	T3K E12K K15Y
999	----K--R------E--	-488	E5K M8R K15E
1000	-Y------------E-D	-488	A2Y K15E F17D
